# An aqueous photo-controlled polymerization under NIR wavelengths: synthesis of polymeric nanoparticles through thick barriers†

**DOI:** 10.1039/d2sc03952d

**Published:** 2022-09-01

**Authors:** Zilong Wu, Wenbo Fang, Chenyu Wu, Nathaniel Corrigan, Tong Zhang, Sihao Xu, Cyrille Boyer

**Affiliations:** Cluster for Advanced Macromolecular Design and Australian Centre for NanoMedicine, School of Chemical Engineering, The University of New South Wales Sydney NSW 2052 Australia cboyer@unsw.edu.au; Qingdao Institute for Theoretical and Computational Sciences, Institute of Frontier and Interdisciplinary Science, Shandong University Qingdao 266237 Shandong P. R. China

## Abstract

We report an aqueous and near-infrared (NIR) light mediated photoinduced reversible addition–fragmentation chain transfer (photo-RAFT) polymerization system using tetrasulfonated zinc phthalocyanine (ZnPcS_4_^−^) as a photocatalyst. Owing to the high catalytic efficiency and excellent oxygen tolerance of this system, well-controlled polyacrylamides, polyacrylates, and polymethacrylates were synthesized at fast rates without requiring deoxygenation. Notably, NIR wavelengths possess enhanced light penetration through non-transparent barriers compared to UV and visible light, allowing high polymerization rates through barriers. Using 6.0 mm pig skin as a barrier, the polymerization rate was only reduced from 0.36 to 0.21 h^−1^, indicating potential for biomedical applications. Furthermore, longer wavelengths (higher *λ*) can be considered an ideal light source for dispersion photopolymerization, especially for the synthesis of large diameter (*d*) nanoparticles, as light scattering is proportional to *d*^6^/*λ*^4^. Therefore, this aqueous photo-RAFT system was applied to photoinduced polymerization-induced self-assembly (photo-PISA), enabling the synthesis of polymeric nanoparticles with various morphologies, including spheres, worms, and vesicles. Taking advantage of high penetration and reduced light scattering of NIR wavelengths, we demonstrate the first syntheses of polymeric nanoparticles with consistent morphologies through thick barriers.

## Abstract 2

Taking advantage of high penetration and reduced light scattering of NIR wavelengths, we demonstrate the syntheses of polymeric nanoparticles with consistent morphologies through thick barriers.

## Graphical abstract

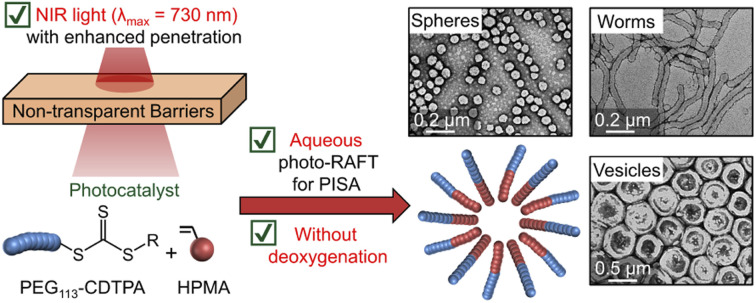

## Introduction

Photoinduced reversible deactivation radical polymerization (photo-RDRP)^1–58^ techniques enable the production of polymers with low dispersity and defined architectures and provide a high degree of spatio–temporal reaction control. As a useful photo-RDRP variant, photoinduced reversible addition–fragmentation chain transfer polymerization (photo-RAFT) polymerization process^26–58^ often uses ppm range photocatalysts (PCs) to activate RAFT agents under visible light without the need for deoxygenation.^48–52^ Although photo-RDRP systems that use visible light (*λ* = 400–700 nm) are well-established, systems regulated by near-infrared light (NIR, *λ* = 700–2500 nm) are still rare.^20–26,43–47^ This is mainly due to the difficulty in using long wavelength, low energy (*E* = *h*/*λ*) irradiation to drive photochemical reactions.^59^ While PCs with NIR light absorption exist, they commonly possess lower redox potentials of excited state (S_1_ and T_1_), resulting in limited catalytic performance and inability to mediate photoinduced electron/energy transfer (PET) processes.^43^ However, compared to shorter wavelengths, NIR light has enhanced penetration through non-transparent barriers,^60^ which can be beneficial for materials engineering and potential biomedical applications. For instance, transdermal photopolymerization has been employed for injectable hydrogel systems, minimizing operative wounds and conferring spatiotemporal control towards gelation.^61–63^

Meanwhile, using aqueous media instead of organic solvents for polymerizations provides economic and environmental benefits, further increasing these systems for bio-applications.^50,61–64^ However, long-wavelength-mediated aqueous photo-RDRP systems have seldom been reported,^17,20,51^ due to the typically low solubility of NIR absorbing catalysts in aqueous media. In 2020, Qiao and co-workers developed an aqueous visible- and NIR-mediated photoinduced electron/energy transfer reversible addition–fragmentation chain transfer (PET-RAFT) polymerization process catalyzed by a self-assembled carboxylated porphyrin photocatalyst.^51^ However, this system suffers from low polymerization rates under NIR irradiation and provided only low monomer conversions without deoxygenation. Recently, the groups of Pan and Pang reported the successful utilization of upconversion nanoparticles as heterogeneous photocatalysts to induce photoinduced atom-transfer radical polymerizations (photo-ATRP) under NIR laser light in aqueous media.^17,20^ Nevertheless, prior deoxygenation and high-intensity light sources were required in these systems to achieve appreciable monomer conversions.

In this study, aqueous photo-RAFT systems catalyzed by tetrasulfonated zinc phthalocyanine (ZnPcS_4_^−^) under NIR light irradiation (*λ*_max_ = 730 nm) were successfully developed. ZnPcS_4_^−^ has been used widely as a photosensitizer in photodynamic therapy (PDT) owing to its absorption of extended wavelengths and excellent solubility in water,^65–70^ however, this is the first example of ZnPcS_4_^−^ as a PC to mediate photopolymerization. These aqueous systems exhibited excellent oxygen tolerance, providing high polymerization rates and polymers with narrow molecular weight distributions (MWDs). Taking advantage of the enhanced penetration of NIR light, photopolymerizations were performed with non-transparent barriers between the light source and the reaction vessel, resulting in high polymerization rates (0.21–0.34 hour^−1^) and well-defined polymers (dispersity (*Đ*) < 1.15). Although photopolymerization through barriers has been demonstrated in previous NIR light mediated RDRP systems,^21–23,44–46,51^ this is the first aqueous system which displays fast polymerization in the presence of thick barriers and does not require deoxygenation. Aqueous media and excellent oxygen tolerance facilitate the potential application of this NIR system in materials engineering and biomedicine.^50,61–64^

In addition, the developed polymerization systems are highly suited for aqueous polymerization-induced self-assembly (PISA) as they do not require deoxygenation, which simplifies the synthetic procedure. More importantly, long wavelength (*λ*) NIR light is an ideal energy source to perform photoinitiated polymerization-induced self-assembly (photo-PISA),^71–82^ especially for synthesizing large (*d*) nanoparticles, as light scattering is directly proportional to *d*^6^/*λ*^4^.^83–87^ Reduced scattering diminishes light intensity gradients in the reaction media, promoting light penetration in colloidal dispersions, which can favor the production of polymeric nanoparticles with more well-defined morphologies. Photo-RAFT mediated aqueous dispersion polymerization of 2-hydroxypropyl methacrylate (HPMA) was conducted using a poly(ethylene glycol) (PEG) functionalized RAFT agent as the first stabilizing block. As a result, nanoparticles with various morphologies, including spheres, worms, and vesicles, were successfully synthesized by simply changing the targeted degree of polymerization (DP). Furthermore, polymeric nanoparticles with consistent morphologies were synthesized *via* photo-PISA through a biological barrier (6.0 mm thick pig skin). To our knowledge, this is the first example of polymeric nanoparticle synthesis through thick barriers using NIR light. Like the barrier-free photo-PISA, we observed a similar evolution of nanoparticle morphologies, *i.e.*, from spheres to vesicles, when a biological barrier was introduced. The successful synthesis of large vesicles (∼200 nm diameter) through thick barriers indicated the high efficiency of this system.

## Results and discussion

### Aqueous photo-RAFT polymerization catalyzed by ZnPcS_4_^−^ under NIR light without deoxygenation

**Scheme 1:**
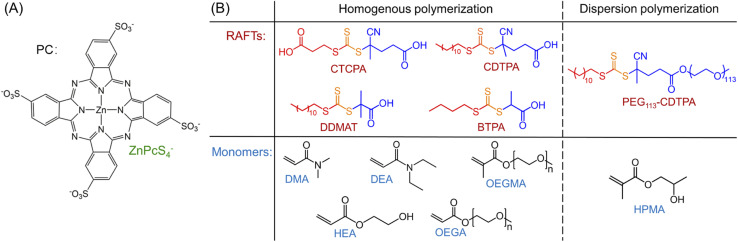
PC, RAFT agents, and monomers used in this work.^a a^(A) PC: tetrasulfonated zinc phthalocyanine (ZnPcS_4_^−^); (B) RAFT agents and monomers used in this work: 4-((((2-carboxyethyl)thio)carbonothioyl)thio)-4-cyanopentanoic acid (CTCPA); 4-cyano-4-[(dodecylsulfanylthiocarbonyl)sulfanyl] pentanoic acid (CDTPA); 2-(dodecylthiocarbonothioylthio)-2-methylpropionic acid (DDMAT); 2-(butylthiocarbonothioylthio)propanoic acid (BTPA); 4-cyano-4-[(dodecylsulfanylthiocarbonyl) sulfanyl] pentanoic acid (CDTPA) functionalized poly(ethylene glycol) (PEG) macromolecular RAFT agent (PEG_113_-CDTPA); *N*,*N*-dimethylacrylamide (DMA); *N*,*N*-diethylacrylamide (DEA); oligo(ethylene glycol) methyl ether methacrylate (OEGMA, *M*_n_ = 300 g mol^−1^); 2-hydroxyethyl acrylate (HEA); oligo(ethylene glycol) methyl ether acrylate (OEGA, *M*_n_ = 480 g mol^−1^); 2-hydroxypropyl methacrylate (HPMA).

Under NIR light (*λ*_max_ = 730 nm) irradiation, tetrasulfonated zinc phthalocyanine (ZnPcS_4_^−^, Scheme 1A and ESI, Fig. S1†) was utilized as a water-soluble photocatalyst (PC) to mediate photo-RAFT polymerization. *N*,*N*-Dimethylacrylamide (DMA) and 4-((((2-carboxyethyl)thio)carbonothioyl)thio)-4-cyanopentanoic acid (CTCPA) were selected as the model monomer and RAFT agent, respectively, owing to their solubility in water (Scheme 1B).

**Scheme 2:**
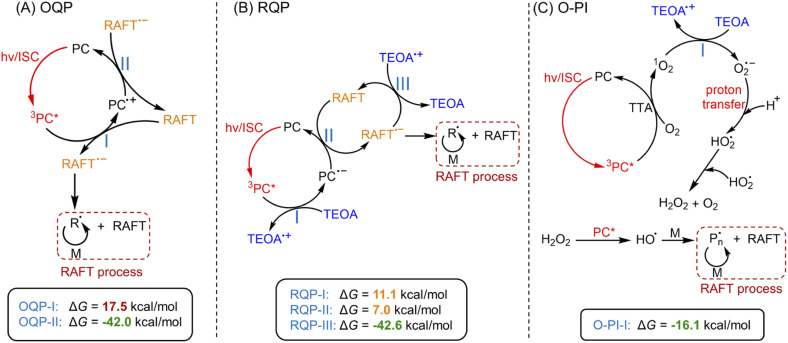
Proposed mechanism of photo-RAFT polymerization catalyzed by ZnPcS_4_^−^*via* three pathways.^a^ Note: ^a^(A) PET-RAFT polymerization *via* an oxidative quenching pathway (OQP) and (B) a reductive quenching pathway (RQP). (C) Photo-RAFT polymerization *via* an oxygen-mediated photoinitiation (O-PI) system. Values indicate the Gibbs free energy change (Δ*G*) of electron transfer reactions in three pathways calculated by DFT (coordinates and energies of PC, RAFT agent, TEOA, and O_2_ were shown in ESI†).

PET-RAFT systems were first tested in the presence of ZnPcS_4_^−^ as PC. As PET-RAFT polymerization can be carried out under two different quenching pathways, namely an oxidative quenching pathway (OQP, Scheme 2A)^43,46,48^ and a reductive quenching pathway (RQP, Scheme 2B).^39,40^ We decided to investigate the efficiency of each pathway in the presence of ZnPcS_4_^−^ under inert atmosphere. Under the OQP, a modest monomer conversion was noted (9% after 10 hours; Table 1, #1), whereas, under the RQP in the presence of triethanolamine (TEOA), a higher monomer conversion was observed (20% after 3 hours; Table 1, #2). In the absence of ZnPcS_4_^−^, both OQP and RQP systems exhibited no monomer conversion (Table 1, #3 and 4). As expected, PET-RAFT polymerization *via* OQP was completely inert without deoxygenation (Table 1, #5), as radical species were quenched by dissolved oxygen. However, surprisingly in the case of RQP in the presence of air, the monomer conversion reached 65% in 3 hours (Table 1, #6). To confirm this surprising result in the presence of TEOA and air, we performed polymerization kinetics with and without deoxygenation. A much higher apparent propagation rate (*k*_p_^app^) (0.36 hour^−1^*versus* 0.08; ESI, Fig. S2†) was determined when the polymerization was conducted without deoxygenation, which suggests a new reaction pathway in the presence of TEOA and oxygen. Control experiments without deoxygenation demonstrated the need for the presence of ZnPcS_4_^−^ (Table 1, #7), TEOA (Table 1, #5), and light (Table 1, #8), as negligible monomer conversions were observed in the absence of either species or light irradiation. Furthermore, in the presence of ZnPcS_4_^−^, TEOA, and oxygen, the control experiment without RAFT agent displayed a modest monomer conversion (18% after 10 hours), suggesting that initiating species can be formed in the absence of RAFT agent (Table 1, #9). This is in contrast to PET-RAFT polymerization *via* RQP (*i.e.*, in the presence of ZnPcS_4_^−^ and TEOA after deoxygenation) where we did not observe monomer conversion in the absence of RAFT agent (Table 1, #10). In the presence of oxygen, photopolymerization displays a different pathway (Table 1, #6 and 9) from PET-RAFT process (Table 1, #2 and 10). Regardless of the conditions employed, PDMA was successfully synthesized in a controlled manner (Table 1, #1, 2, and 6; ESI, Table S1 and Fig. S3†), as indicated by the excellent agreement between theoretical (*M*_n,theo_) and experimental (*M*_n,GPC_) molecular weights, and low dispersity (*Đ*) determined by gel permeation chromatography (GPC).

**Table 1:** Aqueous photo-RAFT polymerizations catalyzed by ZnPcS_4_^−^ under NIR light^a^

#	RAFT agent	PC	Deoxygenation	Reducing agent	Time (hour)	*α* (%)	*k*_p_^app^ (hour^−1^)	*M*_n,theo_ b (g mol^−1^)	*M*_n,GPC_ c (g mol^−1^)	*Đ* c
1	CTCPA	ZnPcS_4_^−^	Yes	—	10	9	0.01	2100	2600	1.16
2	CTCPA	ZnPcS_4_^−^	Yes	TEOA	3	20	0.08	4300	4900	1.12
3	CTCPA	—	Yes	—	10	0	—	—	—	—
4	CTCPA	—	Yes	TEOA	10	0	—	—	—	—
5	CTCPA	ZnPcS_4_^−^	No	—	0	0	—	—	—	—
6	CTCPA	ZnPcS_4_^−^	No	TEOA	3	65	0.36	13 200	13 000	1.09
7	CTCPA	—	No	TEOA	10	0	—	—	—	—
8^d^	CTCPA	ZnPcS_4_^−^	No	TEOA	10	0	—	—	—	—
9	—	ZnPcS_4_^−^	No	TEOA	10	18	—	—	260 700	2.62
10	—	ZnPcS_4_^−^	Yes	TEOA	10	0	—	—	—	—

a Notes: Reactions were performed at room temperature under NIR light (*λ*_max_ = 730 nm; *I* = 60 mW cm^−2^) in water at [DMA]/[water] = 50 (v/v). A fixed reaction stoichiometry of [DMA] : [CTCPA] : [TEOA] : [ZnPcS_4_^−^] = 200 : 1 : 4 : 0.01 (50 ppm ZnPcS_4_^−^ relative to monomer) was used.

b Theoretical molecular weight was calculated using the following equation: *M*_n,theo_ = [*M*]_0_/[RAFT agent]_0_ × MW^M^ × *α* + MW^RAFT agent^, where [*M*]_0_, [RAFT agent]_0_, MW^M^, *α*, and MW^RAFT agent^ correspond to initial monomer concentration, initial RAFT agent concentration, molar mass of monomer, monomer conversion determined by ^1^H NMR, and molar mass of RAFT agent, respectively.

c Molecular weight and dispersity (*Đ*) were determined by GPC analysis (DMAC as eluent) calibrated using PMMA standards.

d Reaction was placed in the dark.

**Fig. 1:**
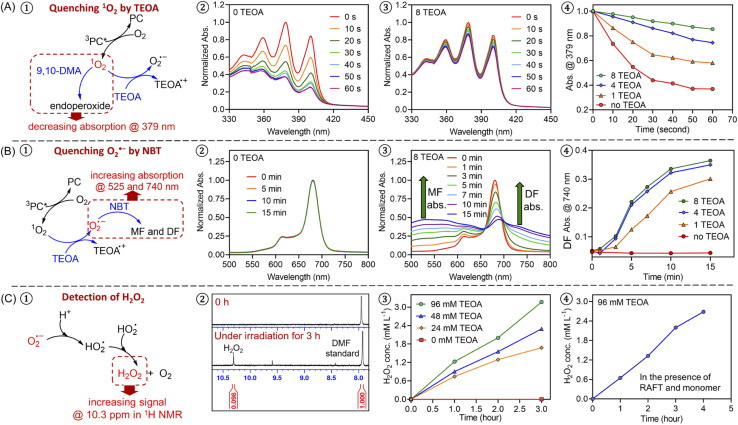
Experimental evidence of an oxygen-mediated photoinitiation (O-PI) system in the presence of TEOA and oxygen. (A) Experiments of quenching singlet oxygen (^1^O_2_) in the presence of 9,10-dimethylanthracene (9,10-DMA, 0.485 mM) with different ratios of TEOA using a stoichiometry of [ZnPcS_4_^−^] : [9,10-DMA] : [TEOA] = 0.002 : 0.02 : (0, 1, 4, and 8) in water/DMF at 50/50 (v/v) under NIR light (*λ*_max_ = 730 nm; *I* = 12 mW cm^−2^); (B) experiments of quenching superoxide (O_2_˙^−^) in the presence of nitrotetrazolium blue chloride (NBT, 1.215 mM) with different ratios of TEOA using a stoichiometry of [ZnPcS_4_^−^] : [TEOA] : [NBT] = 0.002 : (0, 1, 4, and 8) : 0.05 in water/DMF at 50/50 (v/v) under NIR light (*λ*_max_ = 730 nm; *I* = 12 mW cm^−2^); (C) the formation of H_2_O_2_ as indicated by ^1^H NMR spectra (d_6_-DMSO) with increasing irradiation time in the presence of 0.24 mM ZnPcS_4_^−^ and different concentrations of TEOA (0, 24, 48, and 96 mM) without deoxygenation under the irradiation of NIR light (*λ*_max_ = 730 nm; *I* = 60 mW cm^−2^) in water. Experiments were conducted in the absence (#3) and the presence (#4) of monomer and RAFT agent (DMA and CTCPA).

To further investigate the photopolymerization mechanism in the presence of TEOA without deoxygenation, we performed a model reaction in water containing ZnPcS_4_^−^, TEOA, and air under light irradiation. ^1^H NMR spectroscopy was employed to monitor the formation of new species. Interestingly, with increasing irradiation time, we observed the emergence of a new signal at ∼10.3 ppm *via*^1^H NMR (Fig. 1C, #2; ESI, Fig. S4B†), which was attributed to the formation of hydrogen peroxide (H_2_O_2_). The addition of commercial H_2_O_2_ in the reaction medium was carried out resulting in an increased intensity of this signal (ESI, Fig. S4C†), confirming the formation of H_2_O_2_. Notably, a faster generation of H_2_O_2_ was observed in the presence of a higher concentration of TEOA (Fig. 1C, #3; ESI, Fig. S5†), indicating the crucial role of TEOA in H_2_O_2_ formation. In a following experiment, RAFT agent and monomer were added, the signal at 10.3 ppm was also detected (Fig. 1C, #4; ESI, Fig. S6†), showing that RAFT agent or monomer does not interfere with the formation of H_2_O_2_. Having established the formation of H_2_O_2_, we turned our attention to its role in the initiation of this polymerization. Previous publications examining zinc phthalocyanines for photodynamic therapy demonstrated their capability to photosensitize (hydro)peroxides to generate initiating species under light irradiation (ESI, Fig. S7A†).^88,89^ To determine if H_2_O_2_ can be activated by ZnPcS_4_^−^, a small quantity of commercial H_2_O_2_ (24.3 mM) was added to the reaction mixture consisting of ZnPcS_4_^−^, DMA, and CTCPA in water. Under NIR light exposure, we observed 68% monomer conversion after 2 hours (ESI, Fig. S7B,† green line), which indicated that H_2_O_2_ was successfully activated by this system. In the absence of light or ZnPcS_4_^−^, no polymerization was observed (ESI, Fig. S7B,† red points).

Intrigued by the formation of H_2_O_2_, we decided to investigate the mechanism in detail. First, a series of control experiments were carried out to confirm the role of ZnPcS_4_^−^, oxygen and light in the production of H_2_O_2_. After deoxygenation, no formation of H_2_O_2_ was detected after 3 hours in the presence of ZnPcS_4_^−^ and TEOA under NIR light irradiation (ESI, Fig. S4E†). Without deoxygenation, similar results were obtained in the absence of ZnPcS_4_^−^ or light (ESI, Fig. S4D and F†). As reported in previous publications,^90,91^ H_2_O_2_ can be generated from reactive oxygen species (ROS), such as singlet oxygen (^1^O_2_) or superoxide (O_2_˙^−^). Under light irradiation and in the presence of excited ZnPcS_4_^−^ (^3^PC*), oxygen (O_2_) can be transformed into singlet oxygen (^1^O_2_) by triplet–triplet annihilation (TTA; Scheme 2C).^92,93^ To confirm the formation of ^1^O_2_, we performed model experiments in the absence of TEOA and RAFT agents but in the presence of ZnPcS_4_^−^ and 9,10-dimethylanthracene (9,10-DMA) as ^1^O_2_ quencher^94^ (Fig. 1A, #1; ESI, Scheme S1A†) using water and DMF as the solvent. DMF was used to increase the solubility of 9,10-DMA and replace the monomer in our system. Under irradiation, we observed a decrease of 9,10-DMA signals between 330 and 420 nm (Fig. 1A, #2) due to the formation of endoperoxide (ESI, Scheme S1A†), confirming the generation of ^1^O_2_. According to previous reports,^95–97^ we hypothesized that ^1^O_2_ can react with TEOA to yield superoxide (O_2_˙^−^) (Scheme 2C, O-PI-I). To confirm this assumption, we performed model reactions where different concentrations of TEOA were gradually added, and we measured the consumption of 9,10-DMA. Interestingly, with an increasing concentration of TEOA, we noted a significantly slower disappearance of the characteristic peaks of 9,10-DMA (at ∼379 nm), suggesting that ^1^O_2_ was quenched by TEOA (Fig. 1A, #3 and 4; ESI, Fig. S8†). To confirm that O_2_˙^−^ was generated from the electron transfer between TEOA and ^1^O_2_ (Scheme 2C, O-PI-I), quenching experiments of O_2_˙^−^ in the presence of nitrotetrazolium blue chloride (NBT) were performed (Fig. 1B, #1). Indeed, NBT reacts with O_2_˙^−^ (ESI, Scheme S1B†) to yield monoformazan (MF; *λ*_max_ at ∼525 nm) and diformazan (DF; *λ*_max_ at ∼740 nm).^98^ In the absence of TEOA, no absorptions of characteristic MF and DF signals were observed, indicating the absence of O_2_˙^−^ formation (Fig. 1B, #2). In contrast, two characteristic signals of MF and DF appeared when TEOA was added to the mixture, suggesting O_2_˙^−^ generation (Fig. 1B, #3). Additionally, a faster increase in absorption of MF and DF was observed when a higher ratio of TEOA was added (Fig. 1B #4; ESI, Fig. S9†), indicating a more efficient generation of O_2_˙^−^ in this condition which is in accord with the literature.^98^ Subsequently, the protonation of O_2_˙^−^ (Scheme 2C, proton transfer) results in the formation of hydroperoxyl radical (HO_2_˙),^99,100^ which is regarded as a precursor of H_2_O_2_.^90,91^ The accumulation of H_2_O_2_ was confirmed by the presence of a signal at 10.3 ppm determined by ^1^H NMR analysis (Fig. 1C; ESI, Fig. S5 and S6†).

In addition to experimental evidence, a series of density-functional theory (DFT) calculations were performed to calculate the Gibbs free energy change (Δ*G*) for the three different reaction pathways (Scheme 2). The reaction step possessing a lower value of Δ*G* indicates a more favorable electron transfer. Δ*G* values of each reaction involved with electron transfer are listed in Scheme 2. In this work, PET-RAFT polymerization *via* RQP is more favorable than OQP, showing lower values of Δ*G* (11.1 kcal mol^−1^ for RQP-I and 7.0 kcal mol^−1^ for RQP-II *versus* OQP-I = 17.5 kcal mol^−1^). This is in agreement with the faster polymerization rate measured in PET-RAFT polymerization *via* RQP than OQP (0.08 hour^−1^*versus* 0.01 hour^−1^ in ESI, Fig. S2†). Furthermore, Δ*G* of the electron transfer from TEOA to ^1^O_2_ in O-PI pathway was calculated as −16.1 kcal mol^−1^, which is significantly more favorable than the activation steps both in OQP and RQP. This computational result confirms our experimental observation that O-PI displays significantly faster polymerization (ESI, Fig. S2†) than OQP and RQP.

### Optimization experiments of ZnPcS_4_^−^ mediated photo-RAFT system

After investigating the activation mechanism in the presence of O_2_ and TEOA, we turned our attention to the optimization of polymerization kinetics by first varying the TEOA concentrations. Kinetics studies of photopolymerization were investigated with four different TEOA ratios related to the RAFT agent, specifically 1 : 1 (24 mM), 2 : 1 (48 mM), 4 : 1 (96 mM), and 8 : 1 (192 mM) (ESI, Fig. S10 and Table S2†). As expected, photopolymerization with the lowest concentration of TEOA, *i.e.*, 1 : 1 (ESI, Fig. S10, red line; Table S2,† #1), resulted in the slowest *k*_p_^app^ (0.06 hour^−1^) and the longest inhibition period (∼90 min). As TEOA acts as an electron donor for ^1^O_2_ to generate O_2_˙^−^ according to our proposed mechanism (Scheme 2C), the formation and protonation of O_2_˙^−^ are restricted at a low concentration of TEOA, leading to the sluggish formation of H_2_O_2_ (Fig. 1C, #3). As H_2_O_2_ plays a crucial role in this photo-RAFT polymerization system, the slow polymerization rate and long induction period observed in the low concentration of TEOA can be attributed to the slow generation of H_2_O_2_. By increasing the TEOA ratio from 1 : 1 to 2 : 1, the inhibition time was significantly reduced from 90 min to 30 min, and *k*_p_^app^ increased significantly from 0.06 to 0.27 hour^−1^ (ESI, Fig. S10, orange line; Table S2,† #2). When [TEOA] : [RAFT agent] increased to 4 : 1, the performance of polymerization was further improved, exhibiting a shorter induction period of around 15 min and a faster reaction rate of 0.36 hour^−1^ (ESI, Fig. S10, green line; Table S2,† #3). However, as the TEOA ratio was increased to 8 : 1, only a very slight increase in *k*_p_^app^ was observed from 0.36 to 0.38 hour^−1^ (ESI, Fig. S10, blue line; Table S2,† #4), indicating that 4 : 1 can be regarded as the optimized condition.

Subsequently, the concentration of PC in this system was optimized (ESI, Fig. S11 and Table S3†). As expected, photopolymerization with 50 ppm ZnPcS_4_^−^ showed a faster apparent propagation rate (0.36 hour^−1^; ESI, Fig. S11, green line; Table S3,† #2) than 20 ppm (0.24 hour^−1^; ESI, Fig. S11, red line; Table S3,† #1). This result can be explained by the faster production of ^1^O_2_ at a higher concentration of ZnPcS_4_^−^, which favors H_2_O_2_ generation and photoinitiation. However, as ZnPcS_4_^−^ concentration increased further to 100 ppm, the *k*_p_^app^ slightly decreased from 0.36 to 0.33 hour^−1^ (ESI, Fig. S11, blue line; Table S3,† #3), which can be attributed to limiting light penetration in the system due to the absorption of ZnPcS_4_^−^.

To demonstrate the versatility of this PC to activate different RAFT agents, three other RAFT agents, including 4-cyano-4-[(dodecylsulfanylthiocarbonyl)sulfanyl]pentanoic acid (CDTPA), 2-(dodecylthiocarbonothioylthio)-2-methylpropionic acid (DDMAT), and 2-(butylthiocarbonothioylthio)propanoic acid (BTPA) (Scheme 1B), were tested in this aqueous system using a relatively high monomer concentration (50 v% (49 wt%)) in water, to enable the solubilization of these RAFT agents. Compared to BTPA-mediated photopolymerization (ESI, Fig. S12, red line; Table S4,† #4), CDTPA and DDMAT (ESI, Fig. S12, orange and blue lines; Table S4,† #2 and 3) displayed faster polymerization rates and better control. This can be attributed to favorable activation of the tertiary R group of CDTPA and DDMAT than the secondary R group of BTPA.^43,101,102^ Moreover, owing to the better water-solubility of CTCPA than CDTPA and DDMAT, a faster polymerization rate and narrower MWD of synthesized PDMA were also observed in the presence of CTCPA (ESI, Fig. S12A, green line; Table S4,† #1). In summary, the optimal polymerization condition was established using CTCPA as the RAFT agent with [TEOA] : [CTCPA] = 4 : 1 in the presence of 50 ppm ZnPcS_4_^−^.

**Fig. 2:**
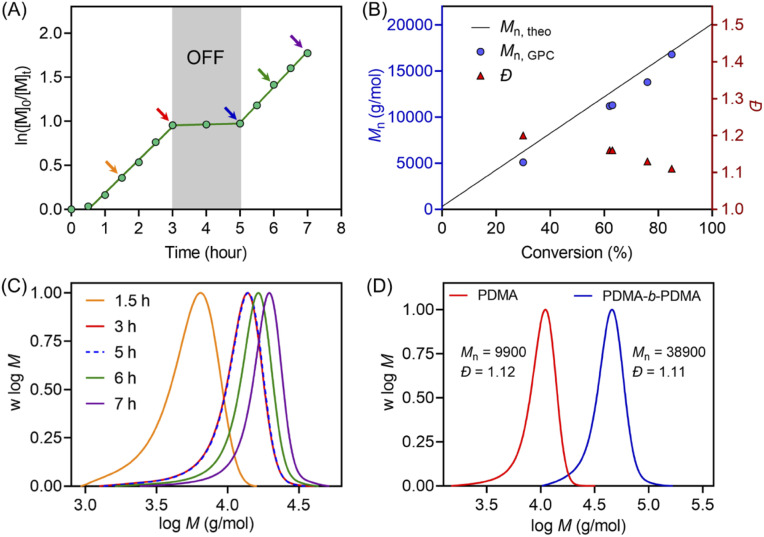
(A) Demonstration of temporal control of ZnPcS_4_^−^ mediated photo-RAFT polymerization regulated by NIR light (*λ*_max_ = 730 nm; *I* = 60 mW cm^−2^) with a reaction stoichiometry of [DMA] : [CTCPA] : [TEOA] : [ZnPcS_4_^−^] = 200 : 1 : 4 : 0.01 at [DMA] : [water] = 50/50 (v/v). (B) Evolution of *M*_n_ and *Đ versus* monomer conversion from the same reaction in (A). (C) Evolution of the normalized MWDs *versus* reaction time using the reaction condition in (A). (D) Normalized MWDs of PDMA and its chain extension with DMA using a reaction stoichiometry of [DMA] : [PDMA macroRAFT agent] : [TEOA] : [ZnPcS_4_^−^] = 400 : 1 : 8 : 0.02 at [DMA] : [water] = 50/50 (v/v).

Temporal control of this aqueous photo-RAFT polymerization was demonstrated (Fig. 2A) by no monomer conversion when the light was turned OFF. Polymerization resumed when the reaction was placed under light irradiation with little change in *k*_p_^app^ (*k*_p_^app^_before_ = 0.38 hour^−1^*versus k*_p_^app^_after_ = 0.40 hour^−1^). In the dark condition, H_2_O_2_ was not activated by ZnPcS_4_^−^, and as a consequence, did not form radicals to initiate the photopolymerization. An excellent agreement between *M*_n,theo_ and *M*_n,GPC_ and a decreasing *Đ* from 1.2 to 1.1 were observed during the polymerization (Fig. 2B). Moreover, the MWDs of synthesized PDMA shifted toward higher molecular weight with increasing irradiation time (Fig. 2C), confirming the controlled character of this aqueous photo-RAFT polymerization. To confirm the maintenance of trithiocarbonate at the end of the polymerization, PDMA was successfully chain extended by adding fresh DMA, leading to clear shifts of MWDs of PDMA-*b*-PDMA (Fig. 2D). Similar results were obtained when poly(oligo(ethylene glycol)methyl ether methacrylate) (POEGMA) was chain extended in the presence of DMA to yield POEGMA-*b*-PDMA block copolymers (ESI, Fig. S13†). Similar to photopolymerizations performed after deoxygenation (ESI, Fig. S14B†), the high end group fidelity of polymers synthesized in the presence of oxygen was also confirmed by ^1^H NMR spectroscopy (ESI, Fig. S14A†). Signals at 5.1 ppm attributed to the CH adjacent to the trithiocarbonate on the R-group side remained in a similar ratio to the CH_3_ protons from the *n*-butyl *Z*-group under both deoxygenated and non-deoxygenated conditions. Subsequently, the degrees of polymerization (DPs) varied from 100 to 800 (ESI, Table S5 and Fig. S15†), resulting in the preparation of PDMA with narrow and symmetric MWDs (*Đ* < 1.2). To investigate the versatility of this system toward various monomers, we decided to expand the family and type of water-soluble monomers (ESI, Table S6 and Fig. S16†). In the presence of ZnPcS_4_^−^, TEOA and CTCPA, *N*,*N*-diethylacrylamide (DEA), 2-hydroxyethyl acrylate (HEA), oligo(ethylene glycol)methyl ether acrylate (OEGA, *M*_n_ = 300 g mol^−1^), and oligo(ethylene glycol)methyl ether methacrylate (OEGMA, *M*_n_ = 480 g mol^−1^) were successfully polymerized in a controlled manner (*Đ* < 1.35) under NIR light irradiation without requiring prior deoxygenation.

### Aqueous photo-RAFT polymerizations through various barriers under NIR light

**Fig. 3:**
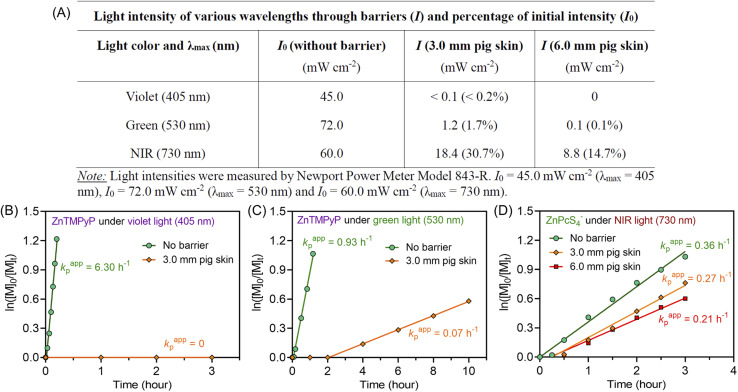
(A) Reduction in light intensity of various wavelengths (405, 530, and 730 nm) after passing through 3.0 and 6.0 mm pig skin. (B and C) ln([*M*]_0_/[*M*]_*t*_) *versus* irradiation time for zinc meso-tetra(*N*-methyl-4-pyridyl)porphine tetrachloride (ZnTMPyP) mediated photo-RAFT polymerization: (B) under violet light (*λ*_max_ = 405 nm; *I* = 45 mW cm^−2^) and (C) green light (*λ*_max_ = 530 nm; *I* = 72 mW cm^−2^) irradiation passing through 3.0 mm pig skin with a reaction stoichiometry of [DMA] : [CTCPA] : [ZnPcS_4_^−^] = 200 : 1 : 0.01 at [DMA]/[water] = 50/50 (v/v) without deoxygenation. (D) ln([*M*]_0_/[*M*]_*t*_) *versus* irradiations time plots of ZnPcS_4_^−^ mediated photo-RAFT polymerization under NIR light (*λ*_max_ = 730 nm; *I* = 60 mW cm^−2^) irradiation after passing through various thicknesses of pig skin with a reaction stoichiometry of [DMA] : [CTCPA] : [TEOA] : [ZnPcS_4_^−^] = 200 : 1 : 4 : 0.01 at [DMA]/[water] = 50/50 (v/v) without deoxygenation.

Notably, compared with UV and visible light, longer wavelengths, such as NIR light, have enhanced penetration through non-transparent materials, making photopolymerization feasible to conduct through thick barriers (ESI, Fig. S17†). A series of photopolymerizations catalyzed by ZnPcS_4_^−^ was performed through various barriers without prior deoxygenation. In the presence of 0.1 and 0.2 mm print paper, although *k*_p_^app^ decreased from 0.36 to 0.26 and 0.21 hour^−1^ (ESI, Fig. S18A†), polymers were successfully synthesized in a controlled manner (ESI, Table S7,† #2 and 3). In addition, biological barriers, such as pig skin, were tested in this system. *K*_p_^app^ declined slightly from 0.36 to 0.34 hour^−1^ (ESI, Fig. S18B,† blue line) when 1.5 mm pig skin was used as the barrier between the NIR LED light and the reaction mixture. When the thickness of pig skin increased further to 3.0 mm, a decrease in *k*_p_^app^ was noted as 0.27 hour^−1^ (Fig. 3D, orange line). Surprisingly, through the 6.0 mm thickness of pig skin, a relatively fast *k*_p_^app^ of 0.21 hour^−1^ was still achieved in this long wavelengths mediated system (Fig. 3D, red line). As a result, polymers were synthesized successfully through barriers with different thicknesses in the presence of oxygen, showing good agreement between *M*_n,theo_ and *M*_n,GPC_ (ESI, Table S7†), narrow MWDs with low dispersity (*Đ* < 1.15; ESI, Fig. S18C and D), and high end group fidelity (ESI, Fig. S19†).

To emphasize the enhanced penetration of NIR light through non-transparent barriers compared to visible light (400–700 nm), the light intensity of various wavelengths (violet light, *λ*_max_ = 405 nm; green light, *λ*_max_ = 530 nm; NIR light *λ*_max_ = 730 nm) were measured before and after passing through barriers with different thicknesses (Fig. 3A). Through 3.0 mm pig skin, the light intensity (*I*) of violet light decreased to 0.2% of the initial value (*I*_0_). While green light was less attenuated than violet light, the light intensity still dropped to only 1.7% of the original *I*_0_ value after passing through 3.0 mm pig skin. In contrast to the limited light penetration of these shorter wavelengths, 730 nm light was able to penetrate through the barriers in appreciable quantities, with 30.7% and 14.7% of the initial light passing through 3.0 and 6.0 mm pig skin, respectively.

To compare the photopolymerization performance between the visible light mediated system and the NIR light mediated system through barriers, zinc meso-tetra(*N*-methyl-4-pyridyl) porphine tetrachloride (ZnTMPyP; Fig. S20†) was employed as a model PC owing to its water solubility and broad absorption in visible regions^50^ (ESI, Table S8 and Fig. S21†). Under violet (*λ*_max_ = 405 nm) and green (*λ*_max_ = 530 nm) light irradiation and in the absence of barriers, photo-RAFT polymerization catalyzed by ZnTMPyP displayed significantly fast polymerization rates (Fig. 3B and C, 6.30 h^−1^ under violet light and 0.93 h^−1^ under green light), which is much faster than NIR light system (0.36 h^−1^) under similar light intensities (*I*_violet_ = 45 mW cm^−2^; *I*_green_ = 72 mW cm^−2^; *I*_NIR_ = 60 mW cm^−2^). However, in the presence of 3.0 mm pig skin as the barrier, polymerization under violet light showed no monomer conversion after 3 hours (Fig. 3B). Meanwhile, a very long induction period (2 hours) and a slow polymerization rate (0.07 h^−1^) were noted when green light was employed (Fig. 3C). In contrast to violet and green light systems, photo-RAFT mediated by NIR light under NIR showed a slight decrease in *k*_p_^app^ from 0.36 to 0.27 h^−1^ (Fig. 3D) when a 3 mm pig skin was introduced.

### Aqueous photo-PISA catalyzed by ZnPcS_4_^−^ under NIR light in the presence of oxygen

Inspired by the monomer versatility and oxygen tolerance, this aqueous photo-RAFT polymerization system was applied to photoinitiated polymerization-induced self-assembly (photo-PISA) to synthesize polymeric nanoparticles. Notably, light scattering limits the light penetration through a dispersion polymerization reaction according to the Rayleigh light scattering equation (proportional to *d*^6^/*λ*^4^), where *d* is the diameter of the particles and *λ* is the wavelength of incident light. Therefore, longer wavelengths of irradiation are more suitable to mediate photoinduced dispersion polymerization, especially for synthesizing large nanoparticles.

**Fig. 4:**
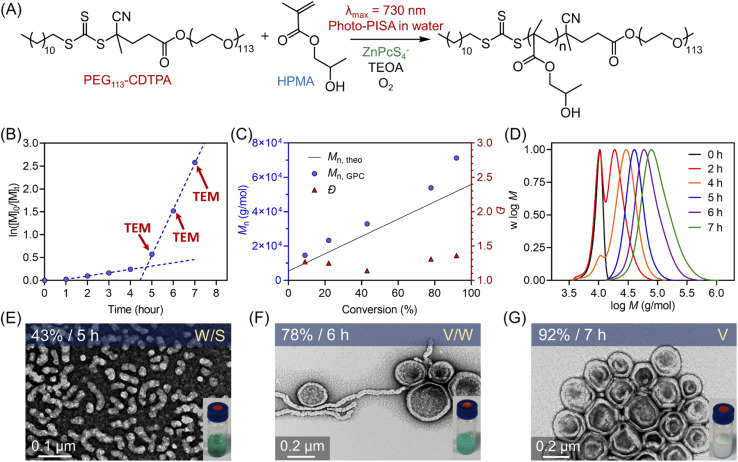
(A) Scheme of ZnPcS_4_^−^ mediated dispersion photopolymerization of HPMA under 730 nm light irradiation (*I* = 60 mW cm^−2^) in the presence of TEOA and oxygen. (B) Kinetics of polymerization of HPMA catalyzed by ZnPcS_4_^−^ under NIR light irradiation using [HPMA] : [PEG_113_-CDTPA] : [TEOA] : [ZnPcS_4_^−^] = 350 : 1 : 4 : 0.02 at a total solids content of 20 wt% (note red arrows indicate the aliquots taken for TEM measurement). (C) Evolution of *M*_n_ and *Đ versus* monomer conversion obtained from the same reaction in (B). (D) Evolution of normalized MWDs *versus* irradiation time from the same reaction in (B). (E–G) TEM images of polymeric nanoparticles obtained at different time points (*t* = 5, 6, and 7 hours).

In this work, PEG_113_-CDTPA macromolecular RAFT agent was used as the first stabilizing block, and the chains were extended in the presence of hydroxypropyl methacrylate (HPMA) as the core-forming monomer in this aqueous photo-RAFT dispersion polymerization (Scheme 1B and Fig. 4A). Optimization experiments with various ratios of TEOA (ESI, Table S9†) were conducted using a 20 wt% solids content of HPMA in water with 1 mL reaction volume in a 2 mL glass vial under NIR light irradiation without prior deoxygenation. Interestingly, in contrast to homogenous polymerization showing good efficiency at [TEOA] : [CTCPA] = 2 (ESI, Table S2,† #2: 53% monomer conversion in 3 hours), a limited monomer conversion (8% monomer conversion in 24 hours) was observed in dispersion photopolymerization using this ratio (ESI, Table S9† #2). This was attributed to the lower monomer content used in dispersion polymerization in contrast to homogenous polymerization (20 wt% *versus* 50 v% (49 wt%)). As [TEOA] : [PEG_113_-CDTPA] increased further to 4 : 1, monomer conversion reached 99% in 24 hours as indicated by ^1^H NMR spectroscopy (ESI, Fig. S22†). GPC analysis confirmed successful chain extension of PEG_113_-CDTPA and the preparation of narrow molecular weight diblock copolymers (ESI, Fig. S23†).

Subsequently, the kinetics study of ZnPcS_4_^−^ mediated photo-RAFT dispersion polymerization of HPMA were investigated at a reaction stoichiometry of [HPMA] : [PEG_113_-CDTPA] : [TEOA] : [ZnPcS_4_^−^] = 350 : 1 : 4 : 0.02 at 20 wt% solids content. As expected, photo-RAFT dispersion polymerization was successfully catalyzed by ZnPcS_4_^−^ without requiring prior deoxygenation under 730 nm light irradiation, although a short inhibition period of 1 hour was observed (Fig. 4A). As reported previously,^49,50,64^ we noted two different regimes: a low *k*_p_^app^ (0.072 hour^−1^) at the starting of the dispersion polymerization (Fig. 4B, 0–4 h), followed by an acceleration of *k*_p_^app^ (1.003 hour^−1^) which was attributed to the formation of nanoparticles (micellization). To confirm this assumption, transmission electron microscopy (TEM) was performed at different time points (*t* = 5, 6, and 7 hours). At *t* = 5 hours, the early stage of the micellization was confirmed by the presence of spherical nanoparticles with ∼20 nm diameter and short worms (Fig. 4E). As the photopolymerization continued, the morphologies of nanoparticles evolved from a mixture of spheres and worms to worms and vesicles (Fig. 4F). At the final point of the kinetics (92% monomer conversion at *t* = 7 hours), pure vesicles with ∼250 nm diameter were observed (Fig. 4G). This evolution in morphology was supported by the apparent change from a transparent solution to a cloudy (milky) solution (Fig. 4E–G, photos at right bottom). In addition, the size distribution of these particles increased with higher monomer conversions, as indicated by dynamic light scattering (DLS) analyses (ESI, Table S10 and Fig. S24†). Moreover, this aqueous dispersion polymerization was performed in a controlled manner, as indicated by the analysis of the diblock copolymers by GPC (Fig. 4C and D). We observed a good agreement between *M*_n,GPC_ and *M*_n,theo_ and relatively low MWD (*Đ* < 1.4). Concerning *Đ*, a decrease was observed first from 1.27 to 1.14 before the micellization (Fig. 4C), which can be attributed to the photoactivation of PEG_113_-CDTPA macroRAFT agent to PEG_113_-*b*-PHPMA with increasing irradiation time as indicated by GPC analyses (Fig. 4D: 0, 2, 4, and 5 hours). After that, *Đ* increased gradually from 1.14 to 1.36 in the phase of dispersion polymerization (Fig. 4C, last three red triangle points). In addition, a clear shift of MWDs towards higher molecular weight with increasing reaction time was observed (Fig. 4D) in this photo-PISA system, indicating the living characters of synthesized polymers.

### Morphological evolution of synthesized nanoparticles with different targeted DPs and solids content

**Fig. 5:**
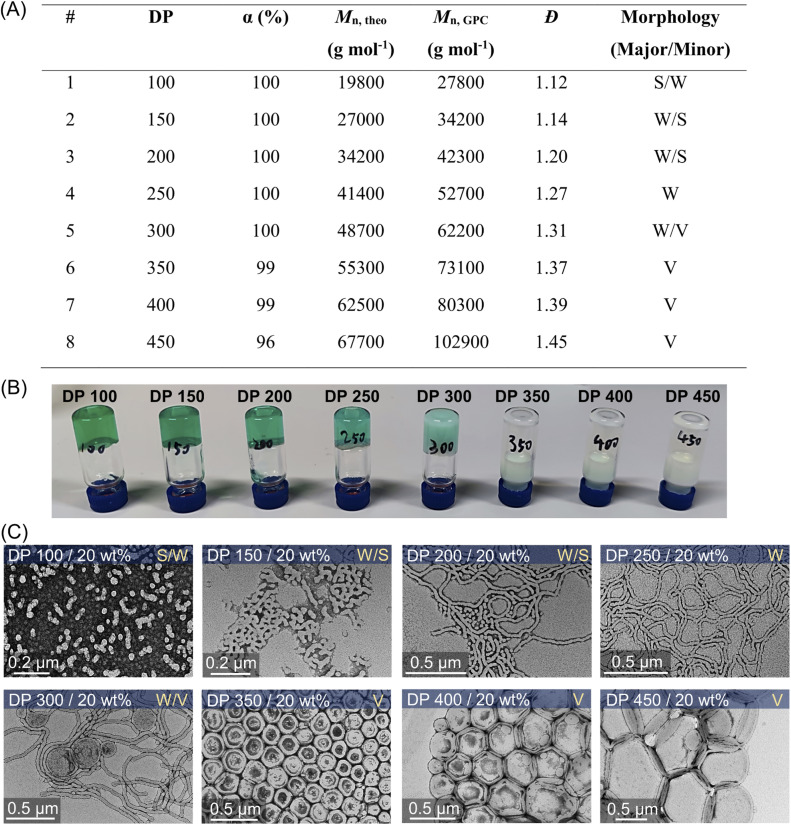
(A) ZnPcS_4_^−^ mediated dispersion photopolymerization of HPMA by varying target DPs from 100 to 450 at 20 wt% total solids content. Polymerizations were conducted under the irradiation of NIR light (*λ*_max_ = 730 nm; 60 mW cm^−2^) for 24 hours without prior deoxygenation in water at a fixed reaction stoichiometry of [PEG_113_-CDTPA] : [TEOA] : [ZnPcS_4_^−^] = 1 : 4 : 0.02. (B) Digital photos of photo-RAFT dispersion polymerization of HPMA with varying target DPs from the same reaction in (A). (C) Corresponding TEM images showing the major and minor morphologies of self-assembled polymeric nanoparticles (S: sphere, W: worms, and V: vesicles).

This aqueous and oxygen tolerant photo-PISA system was investigated further with various targeted DPs at 20 wt% solids content after reaching high monomer conversions (>95%). Overall, dispersion polymerization targeting lower DPs exhibited closer values between *M*_n,GPC_ and *M*_n,theo_ (Fig. 5A), resulting in the synthesis of PEG_113_-*b*-PHPMA with narrower MWDs than polymerization with higher DPs (ESI, Fig. S25†). The reactions ranging from 100 to 300 DPs were significantly viscous, as shown by the digital photos of vials (Fig. 5B), which is likely to correspond to the formation of worms morphologies. Meanwhile, appearances of increasingly cloudy reactions were observed in the reactions with higher targeted DPs, especially for 250, 300, and 350 DPs (Fig. 5B). This indicated that larger-size nanoparticles were prepared with higher DPs, which was also confirmed by DLS measurements (ESI, Table S13, #9–12; Fig. S28C†). Furthermore, TEM was carried out to observe the morphologies for these nanoparticles with different DPs (Fig. 5C). First, a mixture of the majority of spheres and the minority of short worms (S/W) was observed with 100 DP (Fig. 5C, top first). With increasing DPs to 150 and 200, the population of nanoparticles evolved into worms (major) and spheres (minor) (W/S; Fig. 5C, top second and third). Among these two DPs, much longer worms were observed in 200 DP (Fig. 5C, top third) than in 150 DP. Moreover, the morphology of pure worms (W) was observed in the dispersion polymerization with 250 DP (Fig. 5C, top fourth). As the targeted DP increased to 300, a mixed morphology appeared again, consisting of worms and some vesicles (W/V; Fig. 5C, bottom first). As DPs increased further from 350 to 450, we observed pure vesicles with increasing sizes from ∼200 to ∼500 nm (Fig. 5C, bottom last three).

**Fig. 6:**
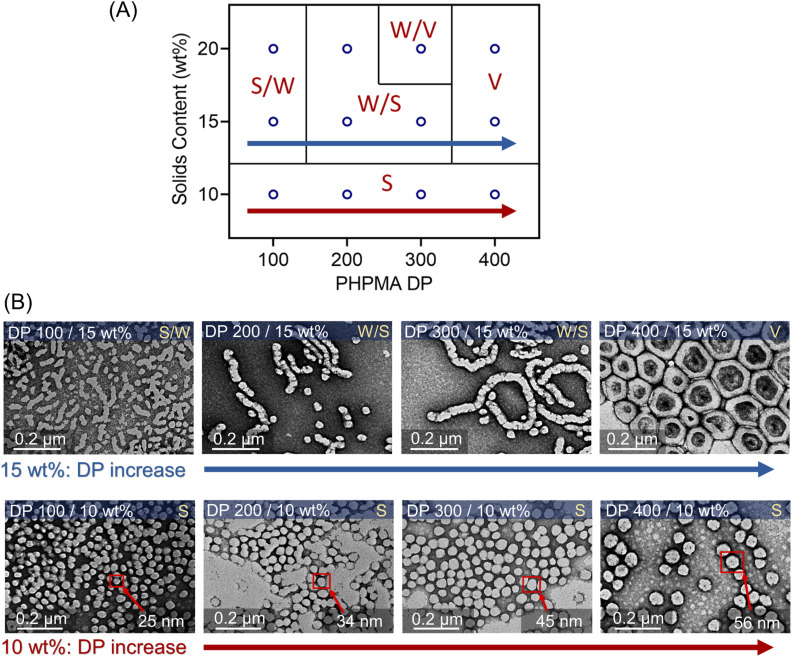
(A) Phase diagram of photo-PISA synthesized by ZnPcS_4_^−^ mediated dispersion photopolymerization of HPMA. Polymerizations were conducted under the irradiation of 730 nm light (*I* = 60 mW cm^−2^) for 24 hours without prior deoxygenation in water at fixed reaction stoichiometries of [PEG_113_-CDTPA] : [TEOA] : [ZnPcS_4_^−^] 1 : 4 : 0.02. (B) TEM images of synthesized polymeric nanoparticles at 10 and 15 wt% solids content (note: TEM images at 20 wt% were presented in Fig. 5C).

The evolution of the morphology was also studied at 10 and 15 wt% solids concentration by varying DPs from 100 to 400. A phase diagram was plotted, reflecting the morphologies of these nanoparticles *versus* various DPs and solids content (Fig. 6A). At 10 wt% solids content, although the morphologies of synthesized PEG_113_-*b*-PHPMA did not show any transformation with increasing DPs, we observed an apparent increase of spherical nanoparticle sizes from ∼25 nm at 100 DP to ∼56 nm at 400 DP (Fig. 6B, bottom row). In contrast to 10 wt%, the change in morphology at 15 wt% solids concentration was similar to 20 wt%, evolving from S/W to V with increasing DPs. On the other hand, there are size differences of synthesized nanoparticles between 15 wt% (Fig. 6B, top row) and 20 wt% (Fig. 5C), which can be easily observed in TEM and DLS analyses (ESI, Table S13 and Fig. S28†). For an example of 400 DP, the diameter of synthesized vesicles at 15 wt% was ∼200 nm, while at 20 wt% solids concentration, the size was ∼400 nm. Overall, a phase diagram was successfully plotted in this photo-PISA system (Fig. 6A), facilitating the nanoparticle synthesis with specific morphologies and sizes under NIR light irradiation. Furthermore, PEG_113_-*b*-PHPMA copolymers forming the nanoparticles displayed narrow MWDs (ESI, Fig. S26 and S27†) and a good correlation between *M*_n,GPC_ and *M*_n,theo_ (ESI, Tables S11 and S12†).

### Synthesis of polymeric nanoparticles through thick barriers under NIR wavelengths

**Fig. 7:**
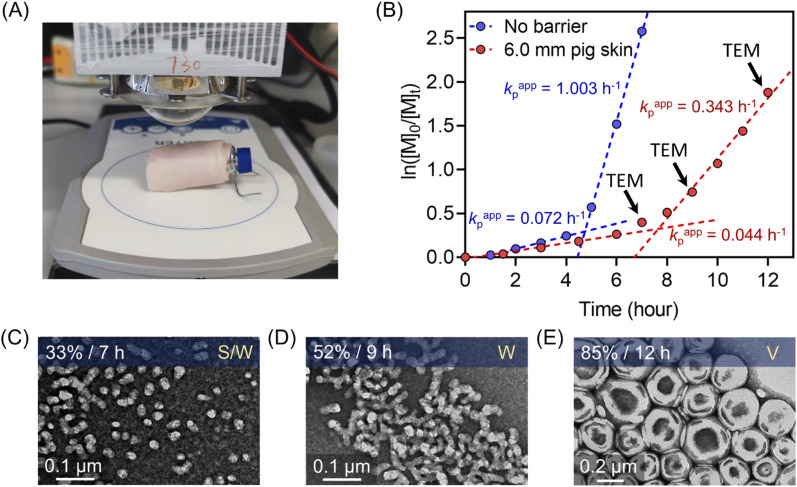
(A) Experimental setup of photo-RAFT dispersion polymerization under the NIR light (*λ*_max_ = 730 nm; 60 mW cm^−2^) irradiation passing through 6.0 mm pig skin. (B) Comparison of dispersion photo polymerization kinetics between without barrier and through 6.0 mm pig skin. (C–E) Evolution of morphologies of polymeric nanoparticles synthesized through 6.0 mm pig skin indicated by corresponding TEM images at different time points (*t* = 7, 9, and 12 hours).

As demonstrated previously in homogenous photopolymerizations, longer wavelengths possess enhanced penetration through non-transparent materials, enabling the synthesis of polymers through thick barriers. Photopolymerization through barriers can be considered more challenging in dispersion polymerization than in the homogenous solution, due to the light scattering of particles impeding the light penetration through the reaction media. As the homogenous photo-RAFT process exhibited high efficiency using 6.0 mm pig skin as the barrier (Fig. 3), this thickness of pig skin was selected in the photo-PISA system (Fig. 7A). Compared to barrier-free photopolymerization, *k*_p_^app^ declined slightly from 0.072 to 0.044 hour^−1^ before micellization (0–7 hours) in the presence of the pig skin barrier (Fig. 7B). After micellization (7–12 hours), *k*_p_^app^ decreased more significantly from 1.003 (without barrier) to 0.343 hour^−1^ (with barrier), which was attributed to the presence of light scattering due to the formation of the nanoparticles. However, the evolution of nanoparticle morphologies was not affected by the inclusion of this barrier. At *t* = 7 hours (33% monomer conversion), PEG_113_-*b*-PHPMA exhibited a mixed morphology consisting of spheres and worms (Fig. 7C), which evolved to pure worms (Fig. 7D) under light irradiation for additional two hours. At 12 hours, TEM indicated the formation of vesicles with ∼200 nm diameter (Fig. 7E). Moreover, this photoinduced dispersion polymerization system enabled the synthesis of well-defined polymers through thick barriers, showing a linear relationship between *M*_n,GPC_ and monomer conversion (ESI, Fig. S29†).

## Conclusion

ZnPcS_4_^−^ was demonstrated as an efficient photocatalyst to mediate aqueous photo-RAFT polymerization under NIR light irradiation, enabling the synthesis of various polymers (polyacrylamide, polyacrylate, and polymethacrylate) in a controlled manner without the need for prior deoxygenation. Owing to the enhanced penetration capabilities afforded by NIR wavelengths, photopolymerizations were successfully conducted through thick barriers at fast polymerization rates in the presence of air. As longer wavelengths display less light scattering, this aqueous and NIR light mediated system was applied in the photo-PISA system, enabling the successful preparation of polymeric nanoparticles with consistent morphologies in water. Moreover, an evolution from spheres to vesicles was observed with increasing monomer conversion in the kinetics study. By plotting a phase diagram of this photo-PISA system, the targeted synthesis of polymeric nanoparticles with specific morphologies and sizes was accessible. Notably, polymeric nanoparticles with precise morphologies were successfully synthesized through 6.0 mm pig skin under NIR light irradiation, owing to enhanced light penetration and reduced light scattering afforded by long wavelengths.

## Data availability

All data associated with this article have been included in the main text and ESI.†

## Author contributions

Z. W. and C. B. conceived the project and designed the experiments. Z. W. carried out the photopolymerizations and characterization. Z. W. and W. F. carried out the synthesis of polymeric nanoparticles. Z. W. carried out the DFT calculations with C. W.’s help. Z. W. carried out the detection of ROS species with T. Z.’s help. Z. W. carried out the TEM with S. X.’s help. Z. W. wrote the original draft with further revisions by N. C. and C. B.

## Conflicts of interest

The authors declare no conflict of interest.

## Supplementary Material

SC-013-D2SC03952D-s001
