# Supplementary material for: An aqueous photo-controlled polymerization under NIR wavelengths: synthesis of polymeric nanoparticles through thick barriers
Source: Chem Sci. 2022 Sep 1;13(39):11519–32. doi: 10.1039/d2sc03952d (PMC9555728; doi:10.1039/d2sc03952d)
Supplement: SC-013-D2SC03952D-s001 [file SC-013-D2SC03952D-s001.pdf]

Electronic Supplementary Material (ESI) for Chemical Science.
This journal is © The Royal Society of Chemistry 2022

Supporting Information

An Aqueous Photo-Controlled Polymerization under NIR Wavelengths: Synthesis of Polymeric Nanoparticles through Thick Barriers

Zilong Wu,[a] Wenbo Fang,[a] Chenyu Wu,[b] Nathaniel Corrigan,[a] Tong Zhang,[a] Sihao Xu,[a] Cyrille Boyer [a,*]

a- *Cluster for Advanced Macromolecular Design and Australian Centre for NanoMedicine, School of Chemical Engineering, The University of New South Wales, Sydney, NSW 2052, Australia; E-mail: cboyer@unsw.edu.au*
b- *Qingdao Institute for Theoretical and Computational Sciences, Institute of Frontier and Interdisciplinary Science, Shandong University, Qingdao 266237 Shandong, P. R. China*

Contents

EXPERIMENTAL SECTION 3

Materials 3

Instrumentation 3

Synthesis of PEG_{113}-CDTPA macroRAFT 4

Experiments of quenching singlet oxygen ($^1O_2$) and superoxide ($O_2^{\bullet-}$) 4

Homogenous photo-RAFT polymerization 5

Photo-RAFT dispersion polymerization 9

ADDITIONAL RESULTS 11

DFT CALCULATIONS 41

Coordinates and energies of photocatalysts 41

Coordinates and energies of RAFT, TEOA, and O_2 46

REFERENCES 49

EXPERIMENTAL SECTION

Materials

N,N-dimethylacrylamide (DMA, 99%), *N,N*-diethylacrylamide (DEA, 99%), 2-hydroxyethyl acrylate (HEA, 96%), oligo(ethylene glycol) methyl ether acrylate (OEGA, 99%) (M_n = 480 g mol^{-1}), and oligo(ethylene glycol) methyl ether methacrylate (OEGMA, 99%) (M_n = 300 g mol^{-1}) were all purchased from Aldrich and deinhibited by passing through a basic alumina column (Ajax Chemical, AR). 2-Hydroxypropyl methacrylate (HPMA, 97%) was purchased from Aldrich and purified by silica column chromatography using ethyl acetate/hexane (1/8) as the eluent. Zinc tetrasulfonated phthalocyanine ($ZnPcS_{4-}$, 95%) was obtained from phthalocyanine tetrasulfonic acid (Frontier Scientific) neutralized with quantitative sodium bicarbonate ($NaHCO_3$, AR). Thiocarbonylthio compounds (RAFT agents), including 4-cyano-4-[(dodecylsulfanylthiocarbonyl)sulfanyl] pentanoic acid (CDTPA, 97%), 4-((((2-carboxyethyl)thio)carbonothioyl)thio)-4-cyanopentanoic acid (CTCPA, 95%), 2-(Butylthiocarbonothioylthio)propanoic acid (BTPA, 95%) were purchased from Boron Molecular while 2-(dodecylthiocarbonothioylthio)-2-methylpropionic acid (DDMAT, 97%) were purchased from Aldrich. Hydrogen peroxide (H_2O_2, 30 wt% in H_2O) was purchased from Chem-Supply. 9,10-Dimethylanthracene (9,10-DMA, 99%), nitrotetrazolium blue chloride (NBT, 90%), triethanolamine (TEOA, 99%), poly(ethylene glycol) methyl ether (PEG_{113}, M_n = 5,000 g mol^{-1}), *N,N'*-dicyclohexylcarbodiimide (DCC, 99%), and 4-(dimethylamino)pyridine (DMAP, 99%) were all purchased from Aldrich and used as received. Zinc meso-tetra(*N*-methyl-4-pyridyl) porphine tetrachloride (ZnTMPyP) was purchased from Frontier Scientific.

Instrumentation

Gel Permeation Chromatography (GPC) was used to characterize synthesized polymer (MWDs, M_n, and $Đ$) with dimethylacetamide (DMAc) as the eluent. The GPC instrument was comprised of a Shimadzu modular system with an autoinjector, a Phenomenex 5.0 μM bead size guard column (50 × 7.5 mm) followed by four Phenomenex 5.0 μM bead size columns (10^5, 10^4, 10^3, and 10^2 Å), and a differential refractive-index detector. The GPC system was calibrated based on the narrow molecular weight distribution of PMMA standards with molecular weights of 200 to 10^6 g mol^{-1}.

Nuclear Magnetic Resonance (NMR) spectroscopy was carried out with Bruker Avance III HD 400 MHz NMR for ^1H using $CDCl_3$ and DMSO-d as solvent. Tetramethylsilane (TMS) was used as a reference. The data was reported as chemical shift (δ) measured in ppm downfield from TMS.

Online Fourier Transform Near-Infrared (FTNIR) was used for monitoring monomer conversion by

calculating the decrement of the vinylic C-H stretching overtone of the monomer at ~ 6200 cm^{-1}. A Bruker IFS 66/S Fourier transform spectrometer equipped with a tungsten halogen lamp, a CaF2 beam splitter, and liquid nitrogen cooled InSb detector was used. The prepared sample was placed in an FTNIR glass cuvette (1 cm × 2 mm) and polymerized under NIR (λ_{max} = 730 nm) LED light irradiation. The sample was manually placed into the cuvette holder, with each spectrum comprising 16 scans with a resolution of 4 cm^{-1} in the region of 8000-4000 cm^{-1}. The measurement time per spectrum was around 10 seconds, and the results from FTNIR were analyzed with OPUS software.

UV-vis Spectroscopy was measured using a CARY 300 spectrophotometer (Varian) using a quartz cuvette (1 cm × 1 cm).

Dynamic Light Scattering (DLS) measurements were performed using a Malvern Zetasizer Ultra and analyzed by ZS Xplorer software. A 4 mW He−Ne laser operating at a wavelength of 633 nm and an avalanche photodiode (APD) detector were used. The scattered light was detected at an angle of 173°.

Transmission Electron Microscopy (TEM) studies of self-assembled block copolymers were conducted at an accelerating voltage of 100 kV (JEOL-1400). The polymerization dispersions were diluted with water to give 0.2 - 0.5 wt% dispersions and deposited onto carbon-coated copper grids. Uranyl acetate staining was applied to all samples.

Photopolymerizations were performed in quartz cuvettes (1 cm × 2 mm) or 2 ml glass vials where the prepared mixtures were irradiated by NIR (λ_{max} = 730 nm), violet (λ_{max} = 405 nm), and green (λ_{max} = 530 nm) LEDs. The light intensity was quantified by Newport 843-R Power Meter.

Synthesis of PEG_{113}-CDTPA macroRAFT agent

CDTPA (0.97 g, 2.4 mmol) in DCM (20 mL) was added to a flask containing PEG_{113} (6 g, 1.2 mmol) and a stirrer bar. Then a solution of DCC (0.5 g, 2.4 mmol) and DMAP (0.029 g, 0.24 mmol) in DCM (10 mL) was added dropwise to the reaction mixture at 0 °C. The flask was sealed with a rubber septum and purged with nitrogen for 30 min at 0 °C. The esterification reaction was allowed to proceed with stirring at room temperature for 48 hours. PEG_{113}-CDTPA macroRAFT agent was collected by filter and repeated precipitation of the reaction mixture in cold diethyl ether.

Experiments of quenching singlet oxygen ($^1O_2$) and superoxide ($O_2^{\bullet-}$)

Experiments of quenching singlet oxygen ($^1O_2$) by 9,10-dimethylanthracene

As an example, experiments of quenching $^1O_2$ with a stoichiometry of [$ZnPcS_4^-$]:[9,10-DMA]:[TEOA] = 0.002:0.02:1 was conducted as follows: A reaction solution consisting of DMF (190 µL), water (170 µL), $ZnPcS_4^-$ (17 µL of 1.113 mM $ZnPcS_4^-$ in water stock solution, 0.0194 µmol), 9,10-dimethylanthracene (9,10-DMA) (10 µL of 19.4 mM 9,10-DMA in DMF stock solution, 0.194 µmol), and TEOA (13 µL of 750 mM TEOA in water stock solution, 9.70 µmol) was prepared in a quartz cuvette (1 cm × 2 mm) and sealed with a rubber septum. Under the irradiation of NIR light (λ_{max} = 730 nm; I = 12 mW cm^{-2}), the characteristic peak of 9,10-DMA (absorption at ~379 nm) was monitored by UV-vis spectroscopy at designated time points. Decreasing absorption of 9,10-DMA reflected the successful $^1O_2$ trapping (**Scheme S1A**). In the presence of higher concentrations of TEOA, slower decreasing rates of 9,10-DMA absorption were observed, indicating $^1O_2$ was also quenched by TEOA.

Experiments of quenching superoxide ($O_2^{\bullet-}$) by nitrotetrazolium blue chloride

As an example, experiments of quenching $O_2^{\bullet-}$ by nitrotetrazolium blue chloride (NBT) with a stoichiometry of [$ZnPcS_4^-$]:[TEOA]:[NBT] = 0.002:4:0.05 was conducted as follows: A reaction solution consisting of water (131 µL), $ZnPcS_4^-$ (17 µL of 1.113 mM $ZnPcS_4^-$ in water stock solution, 0.0194 µmol), TEOA (52 µL of 750 mM TEOA in water stock solution, 38.8 µmol), and NBT (200 µL of 2.43 mM NBT in DMF stock solution, 0.485 µmol) was prepared in a quartz cuvette (1 cm × 2 mm) and sealed with a rubber septum. Under the irradiation of NIR light (λ_{max} = 730 nm; I = 12 mW cm^{-2}), generated $O_2^{\bullet-}$ was quenched by NBT, forming monoformazan (MF; absorption at ~525 nm) and diformazan (DF; absorption at ~740 nm) (**Scheme S1B**). MF and DF. Therefore, increasing absorption of MF and DF reflected the $O_2^{\bullet-}$ trapping. In the presence of TEOA, increasing absorption at ~525 nm and ~740 were observed, indicating $O_2^{\bullet-}$ was generated by TEOA.

Homogenous photo-RAFT polymerization

General procedure for aqueous photo-RAFT polymerizations mediated by $ZnPcS_4^-$ in the absence of oxygen under NIR LED light (PET-RAFT via an oxidative quenching pathway (OQP))

As an example, PET-RAFT polymerization with a reaction stoichiometry of [DMA]:[CTCPA]:[$ZnPcS_4^-$] = 200:1:0.01 was conducted as follows: A reaction solution consisting of water (113 µL), *N, N*-dimethylacrylamide (DMA, 200 µL, 1.941 mmol, 50 vol%), CTCPA (2.98 mg, 9.70 µmol), and $ZnPcS_4^-$ (87 µL of 1.113 mM $ZnPcS_4^-$ in water stock solution, 0.0970 µmol; 50 ppm relative to monomer) was prepared in a quartz cuvette (1 cm × 2 mm) and sealed with a rubber septum. After deoxygenation for 15 mins, the cuvette was placed under a NIR (λ_{max} = 730 nm; I = 60 mW cm^{-2}) LED light at room temperature. The monomer conversion was monitored by online FTNIR by calculating the decrement of the vinylic C-H stretching overtone of the monomer (6100-6220 cm^{-1}) at designated time points. After reaching a certain

monomer conversion, the cuvette was removed from the light source, and aliquots were removed for GPC (DMAc) and ^{1}H NMR analyses.

<u>General procedure for aqueous photo-RAFT polymerizations mediated by $ZnPcS_4^-$ in the absence of oxygen under NIR LED light (PET-RAFT via a reductive quenching pathway (RQP))</u>

As an example, PET-RAFT polymerization with a reaction stoichiometry of [DMA]:[CTCPA]:[$ZnPcS_4^-$] = 200:1:4:0.01 was conducted as follows: A reaction solution consisting of water (108 µL), *N, N*-dimethylacrylamide (DMA, 200 µL, 1.941 mmol, 50 vol%), CTCPA (2.98 mg, 9.70 µmol), $ZnPcS_4^-$ (87 µL of 1.113 mM $ZnPcS_4^-$ in water stock solution, 0.0970 µmol; 50 ppm relative to monomer), and TEOA (5.2 µL, 38.80 µmol) was prepared in a quartz cuvette (1 cm × 2 mm) and sealed with a rubber septum. After deoxygenation for 15 mins, the cuvette was placed under a NIR (λ_{max} = 730 nm; I = 60 mW cm^{-2}) LED light at room temperature. The monomer conversion was monitored by online FTNIR by calculating the decrement of the vinylic C-H stretching overtone of the monomer (6100-6220 cm^{-1}) at designated time points. After reaching relatively high monomer conversion, the cuvette was removed from the light source, and aliquots were removed for GPC (DMAc) and ^{1}H NMR analyses.

<u>General procedure for aqueous photo-RAFT polymerizations mediated by $ZnPcS_4^-$ in the presence of TEOA and oxygen under NIR LED light (via an oxygen-mediated photoinitiation (O-PI))</u>

As an example, photo-RAFT polymerization with a reaction stoichiometry of [DMA]:[CTCPA]:[TEOA]:[$ZnPcS_4^-$] = 200:1:4:0.01 was conducted as follows: A reaction solution consisting of water (108 µL), *N, N*-dimethylacrylamide (DMA, 200 µL, 1.941 mmol, 50 vol%), CTCPA (2.98 mg, 9.70 µmol), $ZnPcS_4^-$ (87 µL of 1.113 mM $ZnPcS_4^-$ in water stock solution, 0.0970 µmol; 50 ppm relative to monomer), and TEOA (5.2 µL, 38.80 µmol) was prepared in a quartz cuvette (1 cm × 2 mm) and sealed with a rubber septum. After that, the cuvette was placed without deoxygenation under a NIR (λ_{max} = 730 nm; I = 60 mW cm^{-2}) LED light at room temperature. The monomer conversion was monitored by online FTNIR by calculating the decrement of the vinylic C-H stretching overtone of the monomer (6100-6220 cm^{-1}) at designated time points. After reaching relatively high monomer conversion, the cuvette was removed from the light source, and aliquots were removed for GPC (DMAc) and ^{1}H NMR analyses.

<u>Procedure for aqueous photo-RAFT polymerizations mediated by $ZnPcS_4^-$ in the presence of H_2O_2 without deoxygenation under NIR LED light</u>

As an example, photo-RAFT polymerization with a reaction stoichiometry of [DMA]:[CTCPA]:[H_2O_2]:[$ZnPcS_4^-$] = 200:1:1:0.01 was conducted as follows: A reaction solution consisting of water (103 µL), *N, N*-dimethylacrylamide (DMA, 200 µL, 1.941 mmol, 50 vol%), CTCPA (2.98 mg, 9.70 µmol), $ZnPcS_4^-$ (87 µL of 1.113 mM $ZnPcS_4^-$ in water stock solution, 0.0970 µmol; 50 ppm relative to monomer), H_2O_2 (10 µL of 970 mM H_2O_2 in water stock solution, 9.70 µmol), and DMF (1.89 mg, 2 µL, 0.066 mmol, added as a ^{1}H NMR internal standard) was prepared in a quartz cuvette (1 cm × 2

mm) and sealed with a rubber septum. After that, the cuvette was placed without deoxygenation under a NIR (λ_{max} = 730 nm; I = 60 mW cm^{-2}) LED light at room temperature. The monomer conversion was monitored by online FTNIR by calculating the decrement of the vinylic C-H stretching overtone of the monomer (6100-6220 cm^{-1}) at designated time points. After reaching relatively high monomer conversion, the cuvette was removed from the light source, and aliquots were removed for GPC (DMAc) and ^{1}H NMR analyses.

<u>General procedure for chain extension mediated by $ZnPcS_4^-$ in presence of TEOA and oxygen under NIR LED light (λ_{max} = 730 nm) in an aqueous solution</u>

In general, the homopolymer was synthesized similarly to the general procedure outlined above. The DP_{target} was reduced to 100 using the first block with a reaction stoichiometry of [DMA]:[CTCPA]:[TEOA]:[$ZnPcS_4^-$] = 100:1:2:0.005. As an example, a PDMA macroRAFT agent was first synthesized in a cuvette containing water (108 µL), *N, N*-dimethylacrylamide (DMA, 200 µL, 1.941 mmol, 50 vol%), CTCPA (5.96 mg, 19.40 µmol), $ZnPcS_4^-$ (87 µL of 1.113 mM $ZnPcS_4^-$ in water stock solution, 0.0970 µmol; 50 ppm relative to monomer), and TEOA (5.2 µL, 38.80 µmol). After that, the cuvette was placed without deoxygenation under a NIR (λ_{max} = 730 nm; I = 60 mW cm^{-2}) LED light at room temperature for 6 hours. The monomer conversion was monitored by online FTNIR by calculating the decrement of the vinylic C-H stretching overtone of the monomer (6100-6220 cm^{-1}). The polymerization was stopped and removed for ^{1}H NMR (93 % monomer conversion) and GPC (M_n = 9900, $Đ$ = 1.12) analyses.

A degree of polymerization (DP) of 400 monomer units was targeted to observe a clear shift in molecular weight distributions (MWDs). For example, for the chain extension of the above PDMA macroRAFT agent with DMA, a reaction stoichiometry of [DMA]:[CTCPA]:[TEOA]:[$ZnPcS_4^-$] = 400:1:8:0.02 was used. The reaction solution consisted of water (8 µL), *N, N*-dimethylacrylamide (DMA, 200 µL, 1.941 mmol, 50 vol%), above crude PDMA macroRAFT agent water solution (100 µL, 4.85 µmol), $ZnPcS_4^-$ (87 µL of 1.113 mM $ZnPcS_4^-$ in water stock solution, 0.0970 µmol; 50 ppm relative to monomer), and TEOA (5.2 µL, 38.80 µmol). After that, the cuvette was placed without deoxygenation under a NIR (λ_{max} = 730 nm; I = 60 mW cm^{-2}) LED light at room temperature for 4 hours. The polymerization was stopped and removed for ^{1}H NMR (83 % monomer conversion) and GPC (M_n = 38900, $Đ$ = 1.11) analyses.

<u>General procedure for aqueous photo-RAFT polymerizations mediated by $ZnPcS_4^-$ in the presence of TEOA and oxygen under NIR LED light (λ_{max} = 730 nm) passing through different barriers</u>

As an example, photo-RAFT polymerization with a reaction stoichiometry of [DMA]:[CTCPA]:[TEOA]:[$ZnPcS_4^-$] = 200:1:4:0.01 was conducted as follows: A reaction solution consisting of water (108 µL), *N, N*-dimethylacrylamide (DMA, 200 µL, 1.941 mmol, 50 vol%), CTCPA (2.98 mg, 9.70 µmol), $ZnPcS_4^-$ (87 µL of 1.113 mM $ZnPcS_4^-$ in water stock solution, 0.0970 µmol; 50 ppm relative to monomer), and TEOA (5.2 µL, 38.80 µmol) was prepared in a quartz cuvette (1 cm × 2 mm) and sealed with a rubber septum. Photopolymerization through barriers was performed by warping the cuvette

with appropriate barriers (**Figure S14**) with the thickness measured by a set of calipers. The barriers used in this test include print paper (~ 0.10 mm), and pig skin (from pig leg with the extra fat removed). Thereafter, the reaction was placed under a NIR (λ_{max} = 730 nm; I = 60 mW cm^{-2}) LED light without deoxygenation at room temperature. The monomer conversion was monitored by online FTNIR by calculating the decrement of the vinylic C-H stretching overtone of the monomer (6100-6220 cm^{-1}) at designated time points. After reaching relatively high monomer conversion, the cuvette was removed from the light source, and aliquots were removed for GPC (DMAc) and ^{1}H NMR analyses.

General procedure for aqueous photo-RAFT polymerizations mediated by ZnTMPyP in the presence of oxygen under violet (λ_{max} = 405 nm) or green (λ_{max} = 530 nm) LED light

As an example, photo-RAFT polymerization with a reaction stoichiometry of [DMA]:[CTCPA]:[$ZnPcS_4^-$] = 200:1:0.01 was conducted as follows: A reaction solution consisting of water (109 μL), *N, N*-dimethylacrylamide (DMA, 200 μL, 1.941 mmol, 50 vol%), CTCPA (2.98 mg, 9.70 μmol), and ZnTMPyP (86 μL of 1.131 mM ZnTMPyP in water stock solution, 0.0970 μmol; 50 ppm relative to monomer) was prepared in a quartz cuvette (1 cm × 2 mm) and sealed with a rubber septum. Subsequently, the cuvette was placed without deoxygenation under a violet (λ_{max} = 405 nm; I_0 = 45 mW cm^{-2}) or a green (λ_{max} = 530 nm; I_0 = 72 mW cm^{-2}) LED light at room temperature. The monomer conversion was monitored by online FTNIR by calculating the decrement of the vinylic C-H stretching overtone of the monomer (6100−6220 cm^{-1}) at designated time points. After reaching relatively high monomer conversion, the cuvette was removed from the light source, and aliquots were removed for GPC (DMAc) and ^{1}H NMR analyses.

General procedure for aqueous photo-RAFT polymerizations mediated by ZnTMPyP in the presence of oxygen under violet (λ_{max} = 405 nm) or green (λ_{max} = 530 nm) LED light passing through 3.0 mm pig skin

As an example, photo-RAFT polymerization with a reaction stoichiometry of [DMA]:[CTCPA]:[$ZnPcS_4^-$] = 200:1:0.01 was conducted as follows: A reaction solution consisting of water (109 μL), *N, N*-dimethylacrylamide (DMA, 200 μL, 1.941 mmol, 50 vol%), CTCPA (2.98 mg, 9.70 μmol), and ZnTMPyP (86 μL of 1.131 mM ZnTMPyP in water stock solution, 0.0970 μmol; 50 ppm relative to monomer) was prepared in a quartz cuvette (1 cm × 2 mm) and sealed with a rubber septum. Photopolymerization through barriers was performed by warping the cuvette with 3.0 mm pig skin. Thereafter, the reaction was placed under a violet (λ_{max} = 405 nm; I_0 = 45 mW cm^{-2}) or a green (λ_{max} = 530 nm; I_0 = 72 mW cm^{-2}) LED light without deoxygenation at room temperature. The monomer conversion was monitored by online FTNIR by calculating the decrement of the vinylic C-H stretching overtone of the monomer (6100-6220 cm^{-1}) at designated time points. After reaching relatively high monomer conversion, the cuvette was removed from the light source, and aliquots were removed for GPC (DMAc) and ^{1}H NMR analyses.

Photo-RAFT dispersion polymerization

Kinetic studies of photo-RAFT dispersion polymerization of HPMA mediated by $ZnPcS_4^-$ in the presence of TEOA and oxygen under NIR LED light (λ_{max} = 730 nm)

For example, kinetics of the photo-RAFT dispersion polymerization of HPMA with a reaction stoichiometry of [HPMA]:[PEG_{113}-CDTPA]:[TEOA]:[$ZnPcS_4^-$] = 350:1:4:0.02 at a total solids content of 20 wt% was conducted as follows: A reaction solution consisting of PEG_{113}-CDTPA (21.61 mg, 4.01 μmol), HPMA (190 μL, 202.54 mg, 1.40 mmol), $ZnPcS_4^-$ (72.10 μg, 0.080 μmol, 72.1 μL of a 1 mg mL^{-1} aqueous stock solution), DMF (9.44 mg, 10 μL, 0.13 mmol, added as the ^1H NMR internal standard), TEOA (2.1 μL, 16.05 μmol) and water (725.8 μL) were prepared in a 2 mL glass vial. A stirrer bar was added to the vial, and the vial was sealed with a cap. After that, the reaction was placed under a NIR (λ_{max} = 730 nm; I = 60 mW cm^{-2}) LED light without deoxygenation on a mixing plate at room temperature. Aliquots were taken for analysis by ^1H NMR, GPC, and TEM at determined time points. Monomer conversions were calculated using ^1H NMR (d_6-DMSO) relative to DMF as an internal standard. Molecular weight values ($M_{n,\,GPC}$) and polymer dispersity ($Đ$) were characterized by GPC (DMAc, PMMA standards). Morphologies of synthesized polymeric nanoparticles were characterized by TEM.

Photo-RAFT dispersion polymerization of HPMA mediated by $ZnPcS_4^-$ in the presence of TEOA and oxygen under NIR LED light (λ_{max} = 730 nm)

For example, photo-RAFT dispersion polymerization of HPMA with a reaction stoichiometry of [HPMA]:[PEG_{113}-CDTPA]:[TEOA]:[$ZnPcS_4^-$] = 300:1:4:0.02 at a total solids content of 10 wt% was conducted as follows: A reaction solution consisting of PEG_{113}-CDTPA (12.48 mg, 2.32 μmol), HPMA (94 μL, 100.20 mg, 0.70 mmol), $ZnPcS_4^-$ (41.6 μg, 0.046 μmol, 41.6 μL of a 1 mg mL^{-1} aqueous stock solution), DMF (9.44 mg, 10 μL, 0.13 mmol, added as a ^1H NMR internal standard), TEOA (1.2 μL, 9.27 μmol) and water (853.2 μL) were prepared in a 2 mL glass vial. A stirrer bar was added to the vial, and the vial was sealed with a cap. After that, the reaction was placed under a NIR (λ_{max} = 730 nm; I = 60 mW cm^{-2}) LED light without deoxygenation on a mixing plate at room temperature. After 24 hours, the reaction was removed from the light source, and aliquots were removed for ^1H NMR, GPC (DMAc), and TEM analyses.

Photo-RAFT dispersion polymerization of HPMA mediated by $ZnPcS_4^-$ under NIR LED light (λ_{max} = 730 nm) passing through 6.0 mm pig skin

Kinetics of the photo-RAFT dispersion polymerization of HPMA with a reaction stoichiometry of [HPMA]:[PEG_{113}-CDTPA]:[TEOA]:[$ZnPcS_4^-$] = 350:1:4:0.02 at a total solids content of 20 wt% was conducted as follows: A reaction solution consisting of PEG_{113}-CDTPA (21.61 mg, 4.01 μmol), HPMA (190 μL, 202.54 mg, 1.40 mmol), $ZnPcS_4^-$ (72.10 μg, 0.080 μmol, 72.1 μL of a 1 mg mL^{-1} aqueous stock solution), DMF (9.44 mg, 10 μL, 0.13 mmol, added as a ^1H NMR internal standard), TEOA (2.1 μL, 16.05 μmol) and water (725.8 μL) were prepared in a 2 mL glass vial. A stirrer bar was added to the vial, and the

vial was sealed with a cap. Photopolymerization through barriers was performed by warping the vial with 6.0 mm pig skin (**Figure 7A**) (the thickness was measured by a set of calipers). After that, the reaction was placed under a NIR (λ_{max} = 730 nm; I = 60 mW cm^{-2}) LED light without deoxygenation on a mixing plate at room temperature. At predetermined time points, aliquots were taken for analysis by ^{1}H NMR, GPC, and TEM. Monomer conversions were calculated using ^{1}H NMR (d_6-DMSO) relative to DMF as an internal standard. Molecular weight values ($M_{n, GPC}$) and polymer dispersity ($Đ$) were characterized by GPC (DMAc, PMMA standards). Morphologies of synthesized polymeric nanoparticles were characterized by TEM.

ADDITIONAL RESULTS

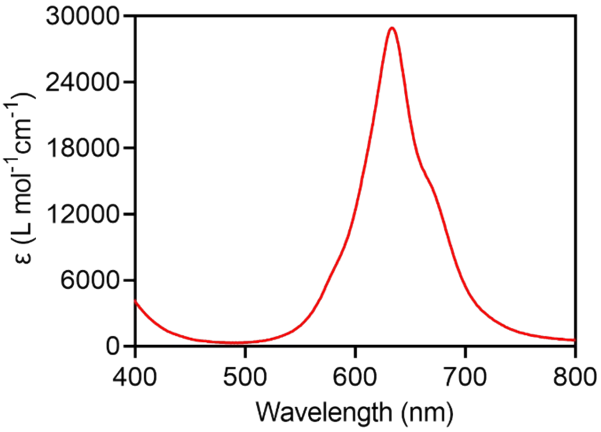

Figure S1. UV-vis spectrum of zinc tetrasulfonated phthalocyanine ($ZnPcS_4^-$) in water.

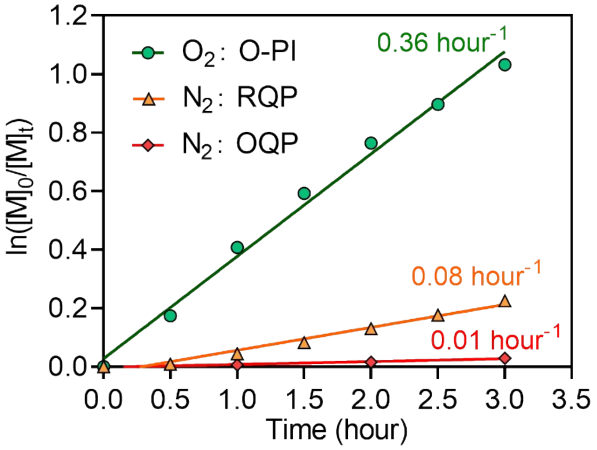

Figure S2. Comparison of the kinetics of photo-RAFT polymerization via three pathways, including PET-RAFT via an oxidative quenching pathway (OQP), PET-RAFT via a reductive quenching pathway (RQP) and an oxygen-mediated photoinitiation (O-PI).

Experimental conditions:

PET-RAFT polymerization via OQP was performed at room temperature under the irradiation of NIR light c after deoxygenation in water at [DMA]/[water] = 50/50 (v/v). A fixed reaction stoichiometry of [DMA]:[CTCPA]:[$ZnPcS_4^-$] = 200:1:0.01 was used.

PET-RAFT polymerization via RQP was performed at room temperature under the irradiation of NIR light (λ_{max} = 730 nm; I = 60 mW cm^{-2}) after deoxygenation in water at [DMA]/[water] = 50/50 (v/v). A fixed reaction stoichiometry of [DMA]:[CTCPA]:[TEOA]:[$ZnPcS_4^-$] = 200:1:4:0.01 was used.

Photo-RAFT polymerization via O-PI was performed at room temperature under the irradiation of NIR light (λ_{max} = 730 nm; I = 60 mW cm^{-2}) without deoxygenation in water at [DMA]/[water] = 50/50 (v/v). A fixed reaction stoichiometry of [DMA]:[CTCPA]:[TEOA]:[$ZnPcS_4^-$] = 200:1:4:0.01 was used.

Table S1. Comparison of $ZnPcS_4^-$ mediated photo-RAFT polymerization in the presence and absence of oxygen under NIR light irradiation.

#	Deoxygenation	Time (hour)	α (%)	k_p^{app} (hour^{-1})	$M_{n, theo}$ (g mol^{-1})	$M_{n, GPC}$ (g mol^{-1})	Đ
1	yes	15	64	0.07	13000	13100	1.10
2	no	3	65	0.36	13200	13000	1.09

Note: Reactions were performed at room temperature under NIR light irradiation (λ_{max} = 730 nm; I = 60 mW cm^{-2}) in water at [DMA]/[water] = 50/50 (v/v). A fixed reaction stoichiometry of [DMA]:[CTCPA]:[TEOA]:[$ZnPcS_4^-$] = 200:1:4:0.01 (50 ppm relative to monomer) was used.

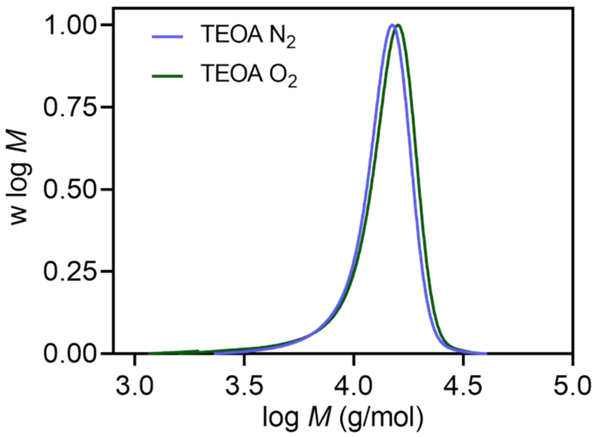

Figure S3. Comparison of MWDs of synthesized PDMA in the presence and absence of oxygen. Reactions were conducted under NIR light (λ_{max} = 730 nm; I = 60 mW cm^{-2}) without (green line) and with deoxygenation (blue line) using a fixed reaction stoichiometry of [DMA]:[CTCPA]:[TEOA]:[$ZnPcS_4^-$] = 200:1:4:0.01.

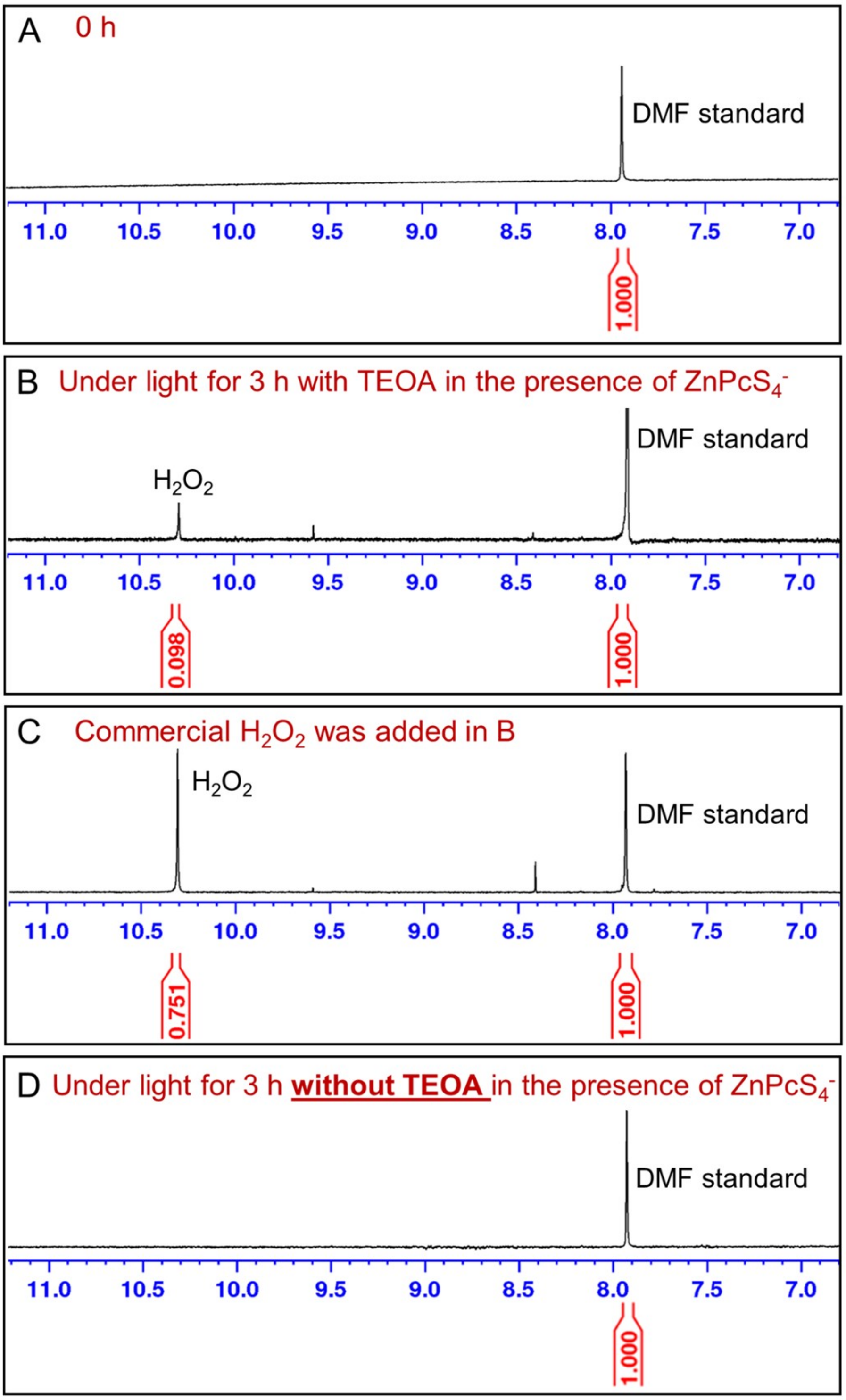
A 0 h
DMF standard
11.0 10.5 10.0 9.5 9.0 8.5 8.0 7.5 7.0
1.000
B Under light for 3 h with TEOA in the presence of ZnPcS$_4^-$
H_2O_2
DMF standard
11.0 10.5 10.0 9.5 9.0 8.5 8.0 7.5 7.0
0.098
1.000
C Commercial H_2O_2 was added in B
H_2O_2
DMF standard
11.0 10.5 10.0 9.5 9.0 8.5 8.0 7.5 7.0
0.751
1.000
D Under light for 3 h **without TEOA** in the presence of ZnPcS$_4^-$
DMF standard
11.0 10.5 10.0 9.5 9.0 8.5 8.0 7.5 7.0
1.000

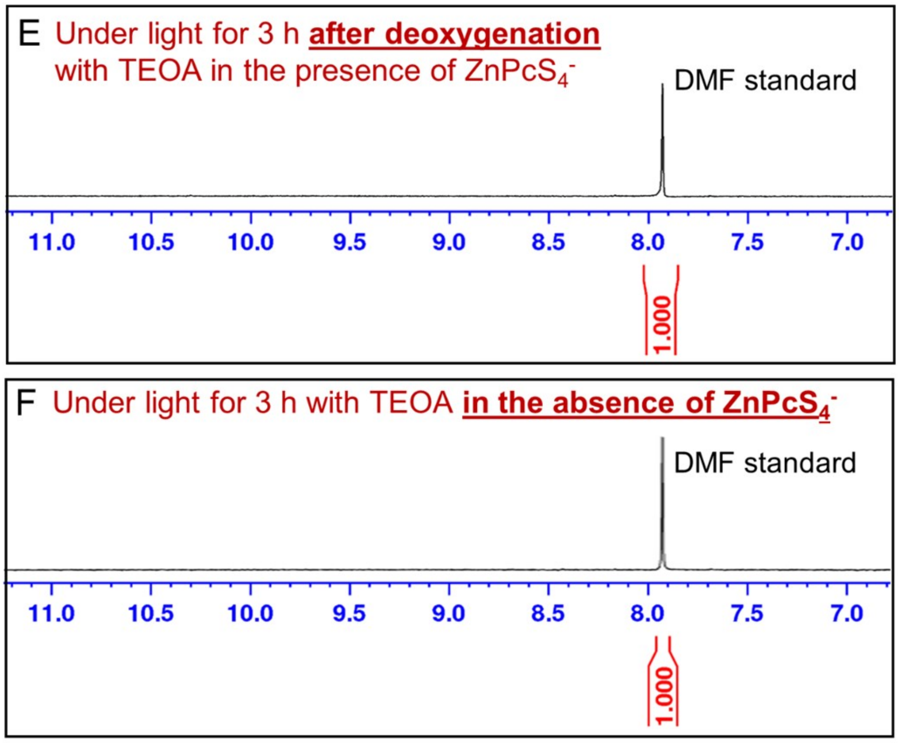

Figure S4. (A and B) The formation of H_2O_2 as indicated by ^{1}H NMR spectra (d_6-DMSO) with increasing irradiation time in the reaction consisting of 0.24 mM $ZnPcS_4^-$ and 96 mM TEOA without deoxygenation. Reaction was performed at room temperature under the irradiation of NIR light (λ_{max} = 730 nm; I = 60 mW cm^{-2}) for 3 hours without deoxygenation. 0.24 mM $ZnPcS_4^-$ and 97 mM TEOA were used, and 64.6 mM DMF was added as the internal standard. (C) 16 mM commercial H_2O_2 was added in (B) to identify the formatting compound at ~ 10.3 ppm. (D) A control experiment in the absence of TEOA but in the presence of $ZnPcS_4^-$ without deoxygenation indicated the absence of H_2O_2 formation under light irradiation for 3 hours. (E) A control experiment in the absence of O_2 (after deoxygenation) but in the presence of $ZnPcS_4^-$ and TEOA indicated the absence of H_2O_2 formation under light irradiation for 3 hours. (F) A control experiment in the absence of $ZnPcS_4^-$ but in the presence of TEOA and without deoxygenation indicated the absence of H_2O_2 formation under light irradiation for 3 hours.

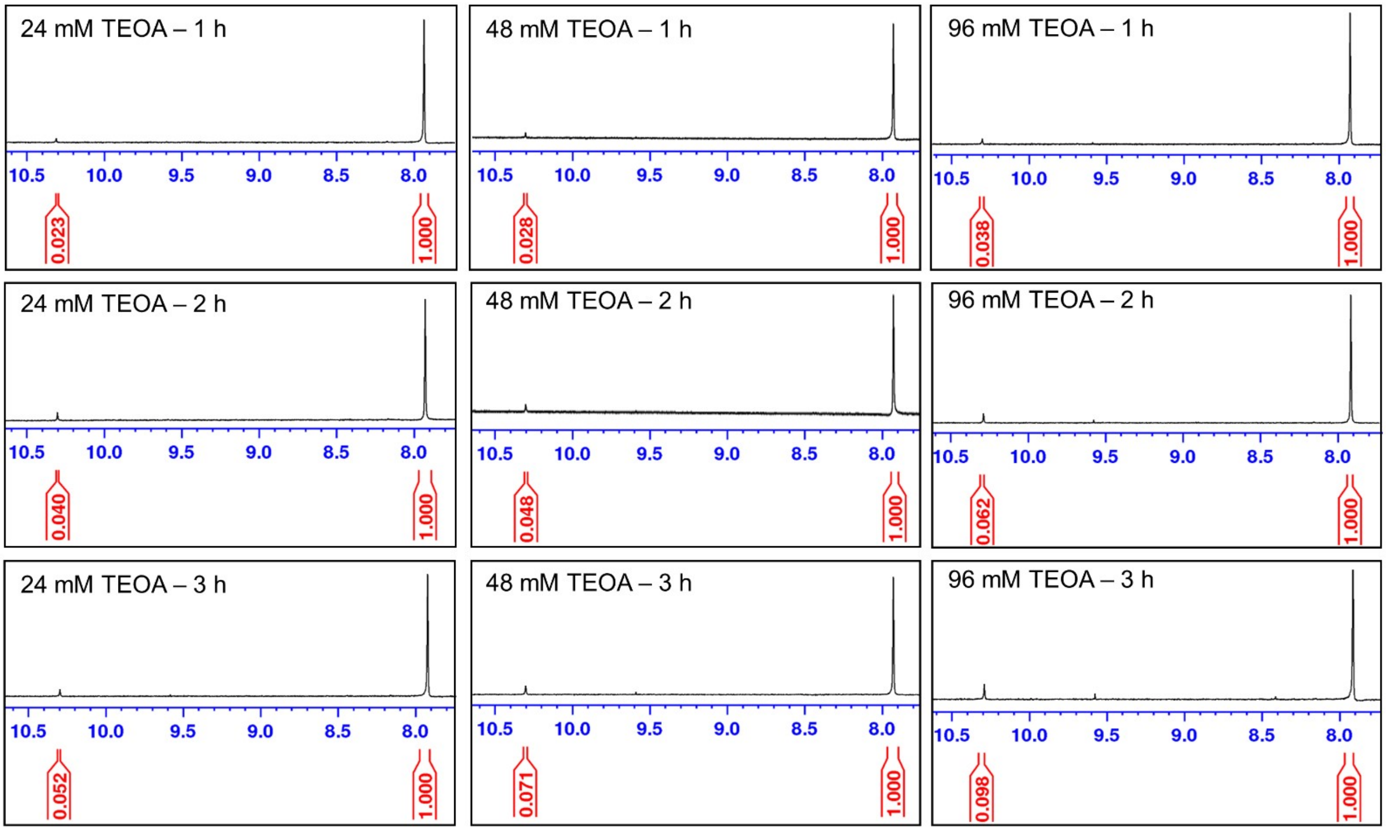

Figure S5. The formation of H_2O_2 was detected by ^1H NMR spectra (d_6-DMSO) with increasing irradiation time (1, 2, and 3 hours) in the presence of different concentrations of TEOA (24, 48, and 96 mM) without deoxygenation. Reactions consisting of 0.24 mM of $ZnPcS_4^-$ and TEOA were performed at room temperature under the irradiation of NIR light (λ_{max} = 730 nm; I = 60 mW cm^{-2}) without deoxygenation in water, and 64.6 mM of DMF was used as internal standard.

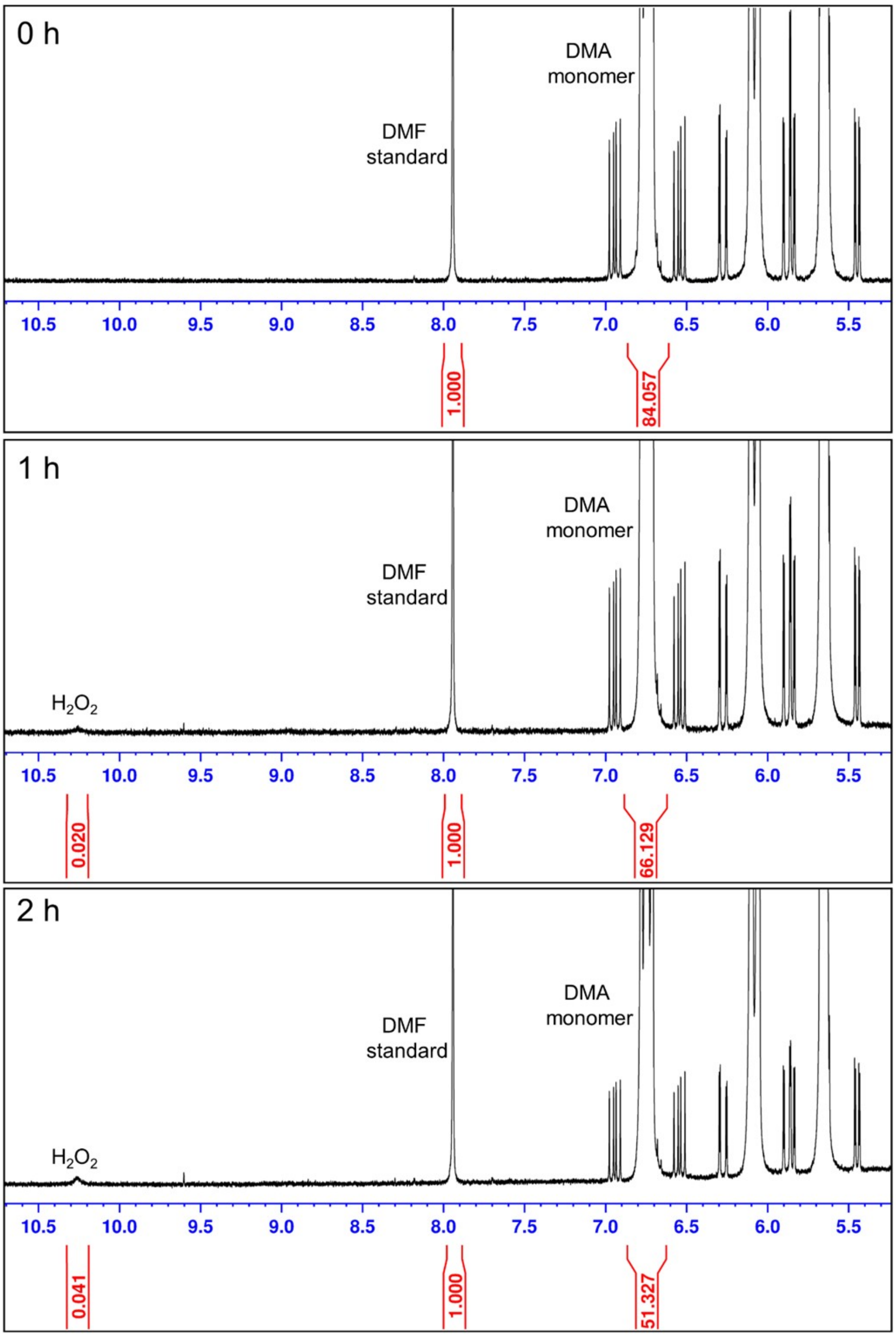
0 h
DMA monomer
DMF standard
10.5 10.0 9.5 9.0 8.5 8.0 7.5 7.0 6.5 6.0 5.5
1.000
84.057
1 h
DMA monomer
DMF standard
H_2O_2
0.020
1.000
66.129
2 h
DMA monomer
DMF standard
H_2O_2
0.041
1.000
51.327

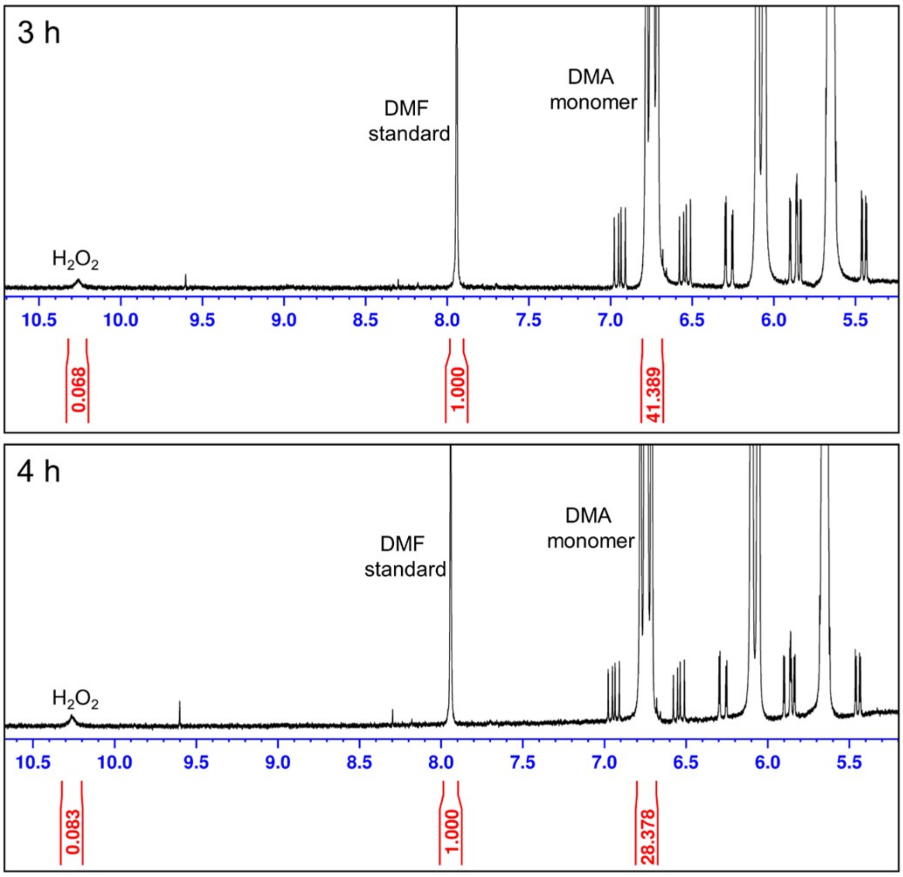

Figure S6. The formation of H_2O_2 was detected by ^{1}H NMR spectra (d_6-DMSO) with increasing irradiation time for $ZnPcS_4^-$ mediated photo-RAFT polymerization in the presence of TEOA without deoxygenation. Photo-RAFT polymerization was performed at room temperature under the irradiation of NIR light (λ_{max} = 730 nm; I = 60 mW cm^{-2}) without deoxygenation in water at [DMA]/[water] = 50/50 (v/v) and 64.6 mM DMF was used as internal standard. A fixed reaction stoichiometry of [DMA]:[CTCPA]:[TEOA]:[$ZnPcS_4^-$] = 200:1:4:0.01 was used.

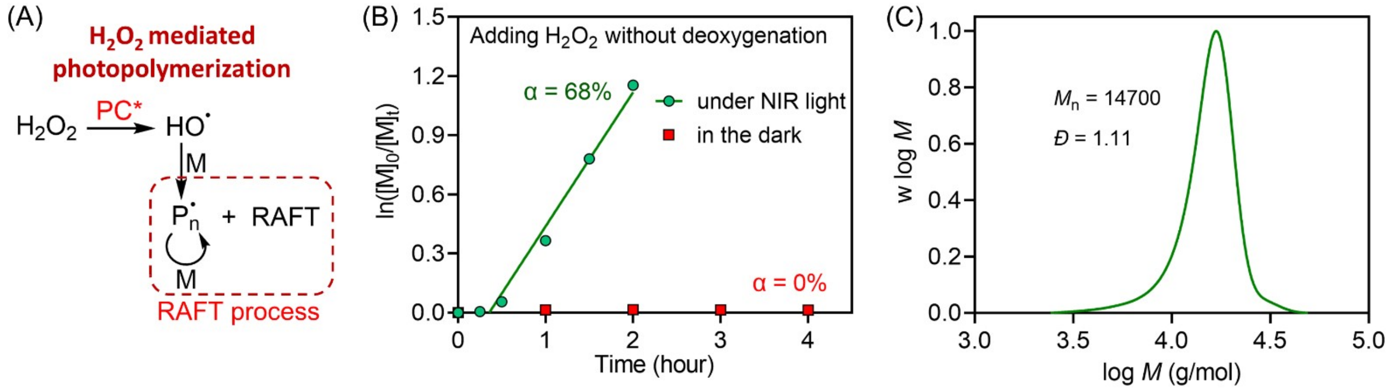

Figure S7. Aqueous photo-RAFT polymerization mediated by $ZnPcS_4^-$ in the presence of 24.3 mM H_2O_2 without deoxygenation under the irradiation of NIR LED light (λ_{max} = 730 nm; I = 60 mW cm^{-2}). Polymerization using a reaction stoichiometry of [DMA]:[CTCPA]:[H_2O_2]:[$ZnPcS_4^-$] = 200:1:1:0.01 was conducted in water at [DMA]/[water] = 50/50 (v/v).

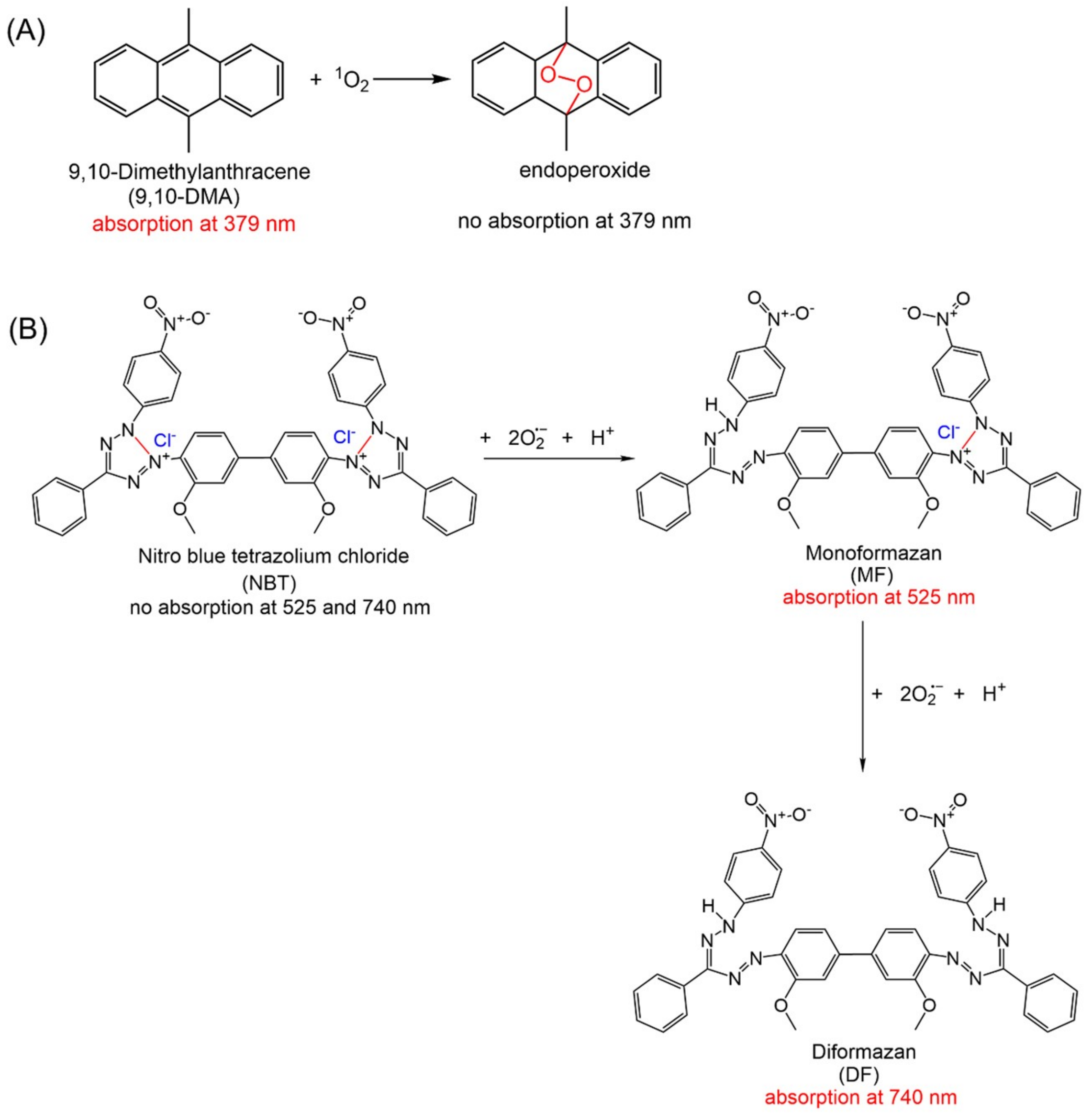

Scheme S1. (A) 9,10-Dimethylanthracene (9,10-DMA; absorption at ~379 nm) reacts with singlet oxygen ($^1O_2$), generating the corresponding endoperoxide.[1] (B) Nitrotetrazolium blue chloride (NBT) reacts with superoxide ($O_2^{\bullet -}$), generating monoformazan (MF; absorption at ~525 nm) and diformazan (DF); absorption at ~740 nm).[2]

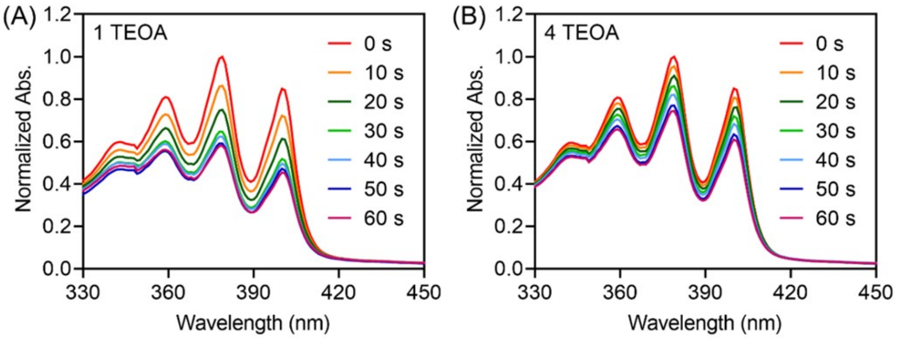

Figure S8. Experiments of quenching singlet oxygen ($^1O_2$) by 0.485 mM 9,10-dimethylanthracene (9,10-DMA) in the presence of different ratios of TEOA using a stoichiometry of [$ZnPcS_4^-$]:[9,10-DMA]:[TEOA] = 0.002:0.02:(1 and 4) in water/DMF at 50/50 (v/v) under NIR light (λ_{max} = 730 nm; I = 12 mW cm^{-2})

Additional discussion: Decreased absorption of 9,10-DMA (at ~379 nm) reflected the successful $^1O_2$ trapping (**Scheme S1A**).

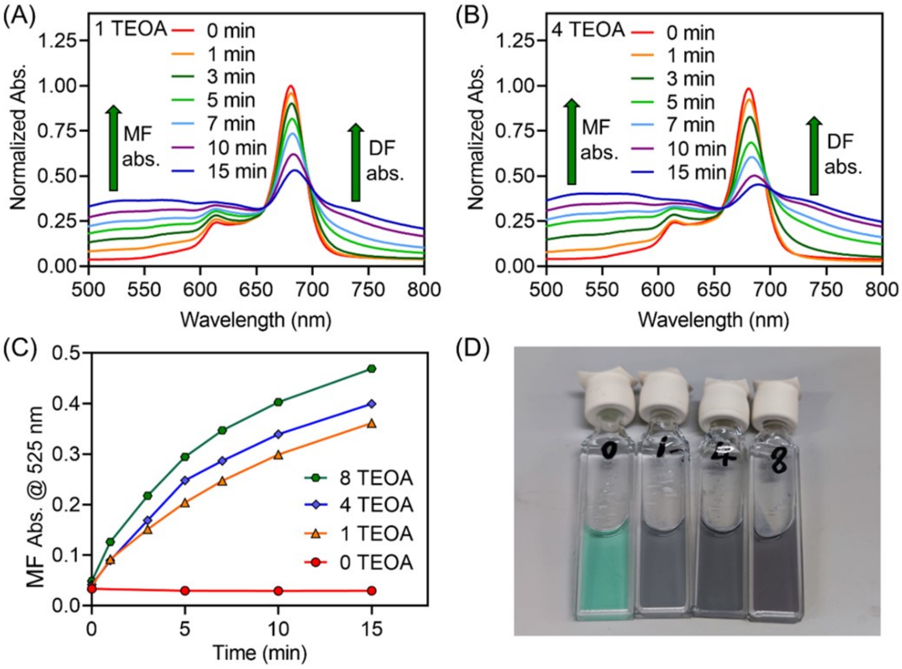

Figure S9. (A−B) Experiments of quenching superoxide ($O_2^{\bullet-}$) by 1.215 mM nitrotetrazolium blue chloride (NBT) in the presence of different ratios of TEOA using a stoichiometry of [$ZnPcS_4^-$]:[TEOA]:[NBT] = 0.002:(1 and 4):0.05 in water/DMF at 50/50 (v/v) under NIR light (λ_{max} = 730 nm; I = 12 mW cm^{-2}); (C) Kinetics of generating MF absorption at ~525 nm in the presence of different ratios of TEOA. (D) Digital photos of $O_2^{\bullet-}$ quenching experiments with different ratios of TEOA under the irradiation of NIR light for 15 mins (the appearance of dark color indicated the formation of MF and DF).

Additional discussion: Generated $O_2^{\bullet-}$ was quenched by NBT, to yield monoformazan (MF; absorption at ~525 nm) and diformazan (DF; absorption at ~740 nm) (**Scheme S1B**).

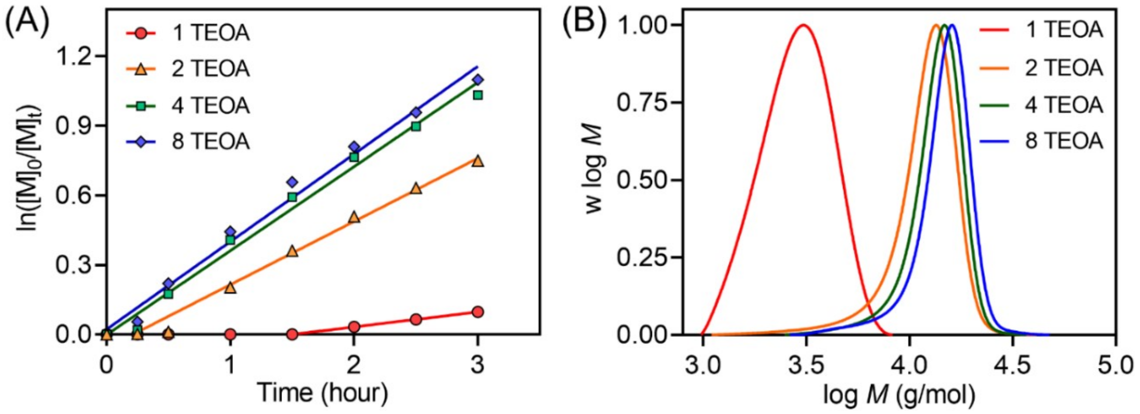

Figure S10. (A) Kinetics and (B) MWDs of $ZnPcS_4^-$ mediated photo-RAFT polymerization using various ratios (1, 2, 4, and 8) of TEOA related to RAFT agent under NIR light irradiation (λ_{max} = 730 nm; I = 60 mW cm^{-2}) for 3 hours.

Table S2. Photo-RAFT polymerization mediated by $ZnPcS_4^-$ with various ratios of TEOA.

#	[TEOA]:[CTCPA]	α (%)	Induction period (min)	k_p^{app} (hour^{-1})	$M_{n, theo}$ (g mol^{-1})	$M_{n, GPC}$ (g mol^{-1})	$Đ$
1	1:1	9	~90	0.06	2100	2700	1.15
2	2:1	53	~30	0.27	10800	11300	1.13
3	4:1	65	~15	0.36	13200	13000	1.09
4	8:1	67	~15	0.38	13600	13600	1.13

Note: Reactions were performed at room temperature under NIR light irradiation (λ_{max} = 730 nm; I = 60 mW cm^{-2}) for 3 hours without prior deoxygenation in water at [DMA]/[water] = 50/50 (v/v). A fixed reaction stoichiometry of [DMA]:[CTCPA]:[$ZnPcS_4^-$] = 200:1:0.01 (50 ppm relative to monomer) was used.

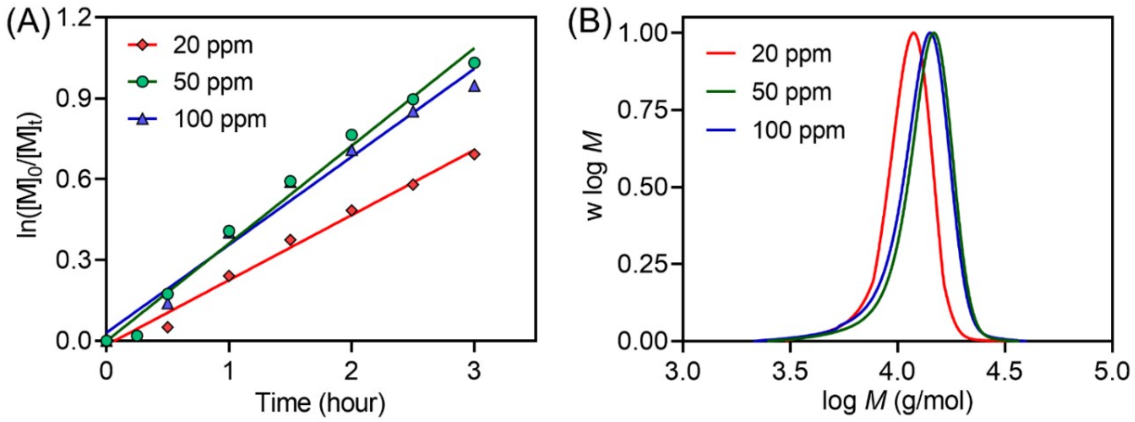

Figure S11. (A) Kinetics and (B) MWDs of $ZnPcS_4^-$ mediated photo-RAFT polymerization with different PC concentrations (20, 50, and 100 ppm related to monomer) under NIR light irradiation.

Table S3. $ZnPcS_4^-$ mediated photo-RAFT polymerization with different PC concentrations under 730 nm light.

#	**PC concentration (ppm)**	**α (%)**	k_p^{app} **(hour^{-1})**	$M_{n, theo}$ **(g mol^{-1})**	$M_{n, GPC}$ **(g mol^{-1})**	$Đ$
1	20	50	0.24	10200	10800	1.07
2	50	65	0.36	13200	13000	1.09
3	100	61	0.33	12400	12300	1.10

<u>Note:</u> were performed at room temperature under the irradiation of NIR light (λ_{max} = 730 nm; I = 60 mW cm^{-2}) for 3 hours without prior deoxygenation in water at [DMA]/[water] = 50/50 (v/v). A fixed reaction stoichiometry of [DMA]:[CTCPA]:[TEOA] = 200:1:4 was used.

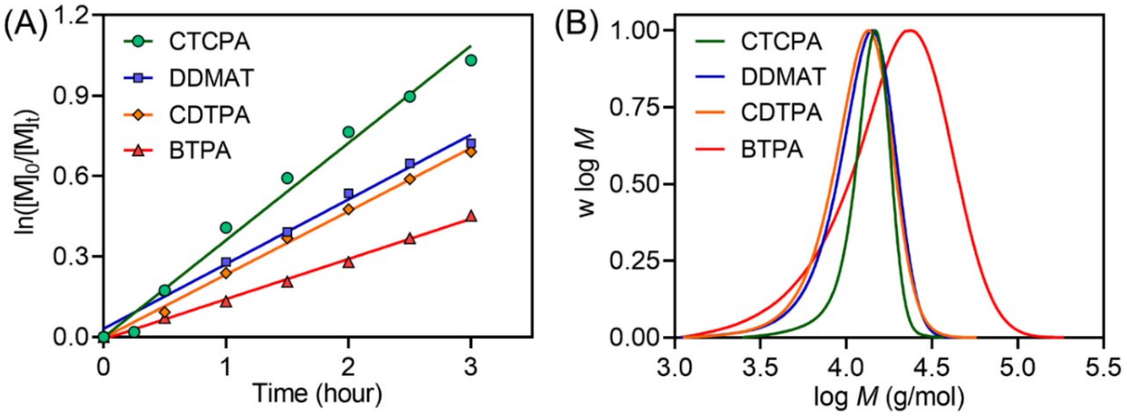

Figure S12. (A) Kinetics and (B) MWDs of $ZnPcS_4^{-}$ mediated photo-RAFT polymerization with different RAFT agents under NIR light irradiation (λ_{max} = 730 nm; I = 60 mW cm^{-2}).

Table S4. $ZnPcS_4^{-}$ mediated photo-RAFT polymerization using different RAFT agents under NIR light.

#	RAFT agent	α (%)	k_p^{app} (hour^{-1})	$M_{n, theo}$ (g mol^{-1})	$M_{n, GPC}$ (g mol^{-1})	*Đ*
1	CTCPA	65	0.36	13200	13000	1.09
2	CDTPA	50	0.23	10300	11000	1.20
3	DDMAT	51	0.24	10500	11400	1.20
4	BTPA	36	0.15	7400	15000	1.59

Note: Reactions were performed at room temperature under the irradiation of NIR light (λ_{max} = 730 nm; I = 60 mW cm^{-2}) for 3 hours without prior deoxygenation in water at [DMA]/[water] = 50/50 (v/v). A fixed reaction stoichiometry of [DMA]:[RAFT agent]:[TEOA]:[$ZnPcS_4^{-}$] = 200:1:4:0.01 (50 ppm relative to monomer) was used.

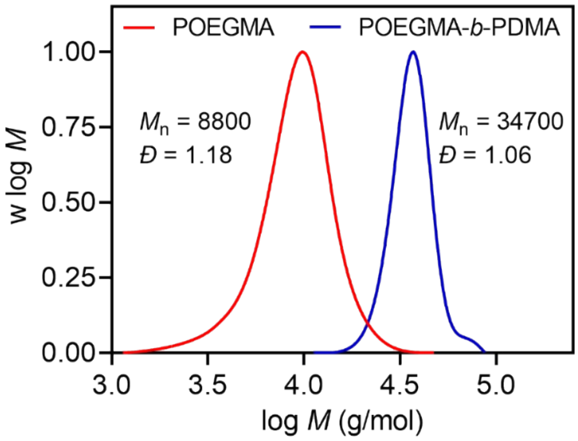

Figure S13. Normalized MWDs of POEGMA and its chain extension with DMA using a reaction stoichiometry of [DMA]:[macroRAFT agent]:[TEOA]:[$ZnPcS_4^{-}$] = 400:1:4:0.02 in water under the NIR light irradiation (λ_{max} = 730 nm; I = 60 mW cm^{-2}).

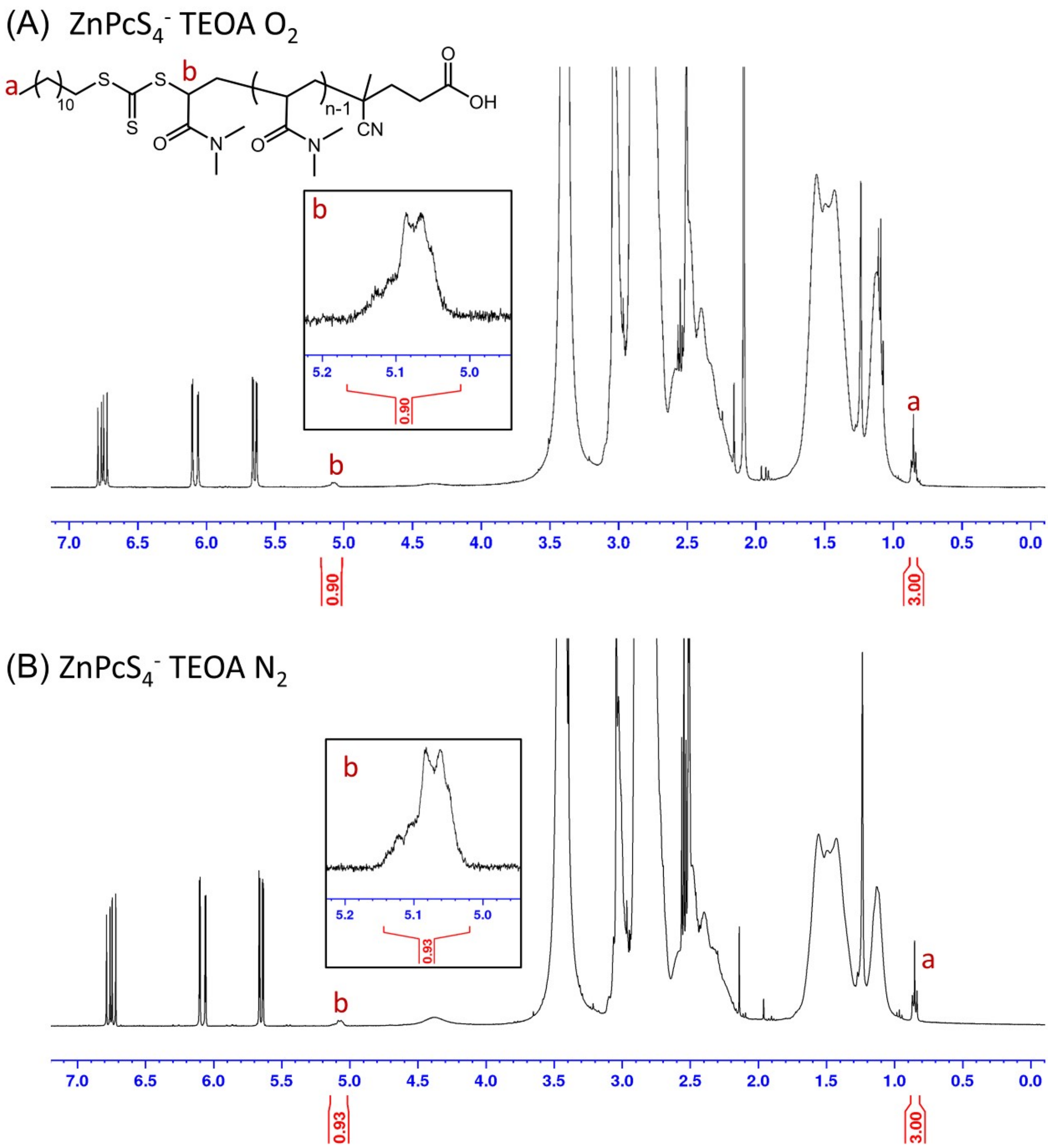

Figure S14. ^{1}H NMR spectroscopy of PDMA synthesized using CDTPA as the RAFT agent. Reactions were performed at room temperature under the irradiation of NIR light (λ_{max} = 730 nm; I = 60 mW cm^{-2}) (A) without prior deoxygenation (monomer conversion = 95%) and (B) after deoxygenation (monomer conversion = 93%) in water at [DMA]/[water] = 50/50 (v/v) using a fixed reaction stoichiometry of [DMA]:[CDTPA]:[TEOA]:[$ZnPcS_4^-$] = 100:1:4:0.005 (50 ppm relative to monomer).

Table S5. $ZnPcS_4^-$ mediated photo-RAFT polymerization with different targeted DPs under the NIR light irradiation.

	**DP**	**α (%)**	$M_{n, theo}$ **(g mol⁻¹)**	$M_{n, GPC}$ **(g mol⁻¹)**	**Đ**
1	100	68	7000	7000	1.14
2	200	84	17000	16800	1.11
3	400	80	32000	26700	1.09
4	800	57	45500	38400	1.13

Note: Reactions were performed at room temperature under the irradiation of NIR light (λ_{max} = 730 nm; I = 60 mW cm^{-2}) for 5 hours without prior deoxygenation in water at [DMA]/[water] = 50/50 (v/v). A fixed reaction stoichiometry of [CTCPA]:[TEOA]:[$ZnPcS_4^-$] = 1:4:0.01 (50 ppm relative to monomer) was used.

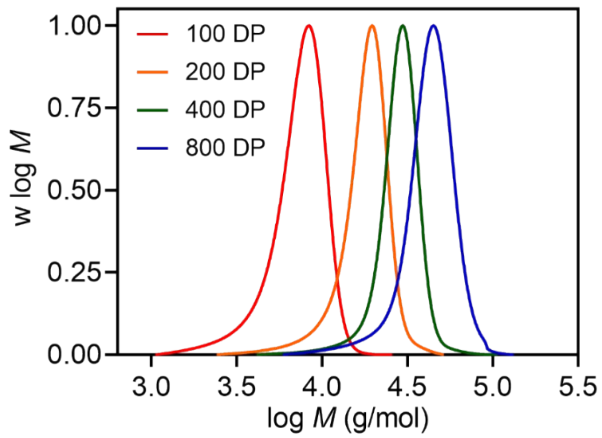

Figure S15. MWDs of $ZnPcS_4^-$ mediated photo-RAFT polymerization with different targeted DPs (100, 200, 400, and 800) under the NIR light irradiation for 5 hours.

Table S6. $ZnPcS_4^-$ mediated photo-RAFT polymerization with various monomers.

#	**Monomer**	**α (%)**	$M_{n,\,theo}$ (g mol^{-1})	$M_{n,\,GPC}$ (g mol^{-1})	**Đ**
1	DEA	53	13800	10600	1.21
2	HEA	51	12100	9500	1.25
3[a]	OEGA	77	37300	23600	1.33
4[a]	OEGMA	72	21900	16100	1.26

Note: *N,N*-diethylacrylamide (DEA), 2-hydroxyethyl acrylate (HEA), Oligo(ethylene glycol) methyl ether acrylate (OEGA, M_n = 300 g mol^{-1}) and Oligo(ethylene glycol) methyl ether methacrylate (OEGMA, M_n = 480 g mol^{-1}) were used as monomers in this work. Reactions were performed at room temperature under the irradiation of NIR light (λ_{max} = 730 nm; I = 60 mW cm^{-2}) for 3 hours without prior deoxygenation in water at [monomer]/[water] = 50/50 (v/v). A fixed reaction stoichiometry of [monomer]:[CTCPA]:[TEOA]:[$ZnPcS_4^-$] = 200:1:4:0.01 (50 ppm relative to monomer) was used.

[a]A reaction stoichiometry of [monomer]:[CTCPA]:[TEOA]:[$ZnPcS_4^-$] = 100:1:2:0.05 (50 ppm relative to monomer) was used.

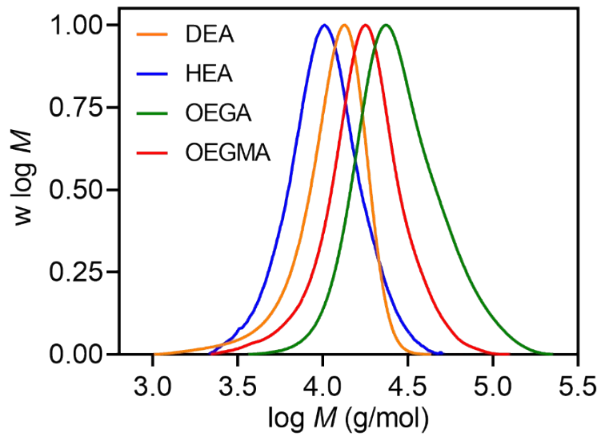

Figure S16. Normalized MWDs of $ZnPcS_4^-$ mediated photo-RAFT polymerization using various monomers, including acrylamide, acrylate, and methacrylate.

Note: *N,N*-diethylacrylamide (DEA), 2-hydroxyethyl acrylate (HEA), oligo(ethylene glycol) methyl ether acrylate (OEGA, M_n = 300 g mol^{-1}) and oligo(ethylene glycol) methyl ether methacrylate (OEGMA, M_n = 480 g mol^{-1}).

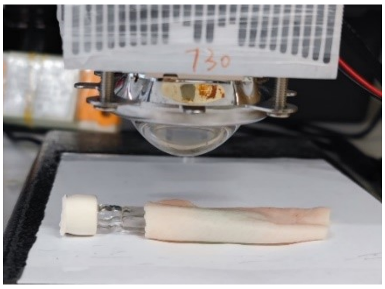

Figure S17. Experimental set-up of photo-RAFT polymerization catalyzed by $ZnPcS_4^-$ under NIR light (λ_{max} = 730 nm; I = 60 mW cm^{-2}) irradiation passing through 1.5 mm pig skin.

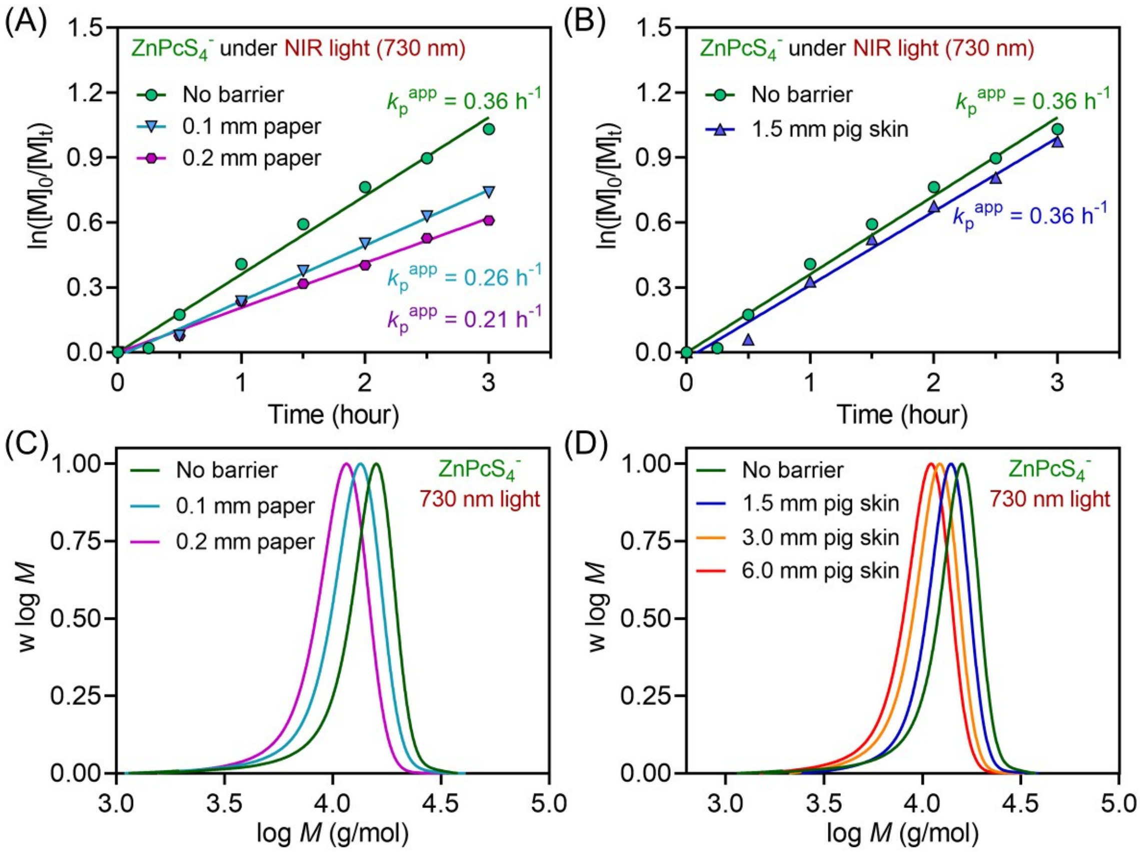

Figure S18. (A−B) ln([M]$_0$/[M]$_t$) *versus* irradiation time plots of $ZnPcS_4^-$ mediated photo-RAFT under NIR light (λ_{max} = 730 nm; I = 60 mW cm^{-2}) irradiation passing through various thicknesses of (A) paper and (B) pig skin with a reaction stoichiometry of [DMA]:[CTCPA]:[TEOA]:[$ZnPcS_4^-$] = 200:1:4:0.01 at [DMA]/[water] = 50/50 (v/v) without deoxygenation. (C−D) MWDs of synthesized PDMA by $ZnPcS_4^-$ mediated photo-RAFT under NIR light (λ_{max} = 730 nm; I = 60 mW cm^{-2}) irradiation for 3 hours passing through various thicknesses of (C) paper and (D) pig skin.

Table S7. $ZnPcS_4^-$ mediated photo-RAFT polymerization under the NIR light irradiation passing through various barriers.

#	Barrier	α (%)	k_p^{app} (hour^{-1})	$M_{n,\,theo}$ (g mol^{-1})	$M_{n,\,GPC}$ (g mol^{-1})	$Đ$
1	no	65	0.36	13200	13000	1.09
2	0.1 mm paper	51	0.26	10400	10900	1.13
3	0.2 mm paper	44	0.21	9000	9600	1.12
4	1.5 mm pig skin	62	0.34	12600	12300	1.09
5	3.0 mm pig skin	53	0.27	10800	10600	1.10
6	6.0 mm pig skin	45	0.21	9200	9100	1.11

Note: Reactions were irradiated by NIR light (λ_{max} = 730 nm; I = 60 mW cm^{-2}) passing through various barriers for 3 hours at room temperature without prior deoxygenation in water at [DMA]/[water] = 50/50 (v/v). A fixed reaction stoichiometry of [DMA]:[CTCPA]:[TEOA]:[$ZnPcS_4^-$] = 200:1:4:0.01 (50 ppm relative to monomer) was used.

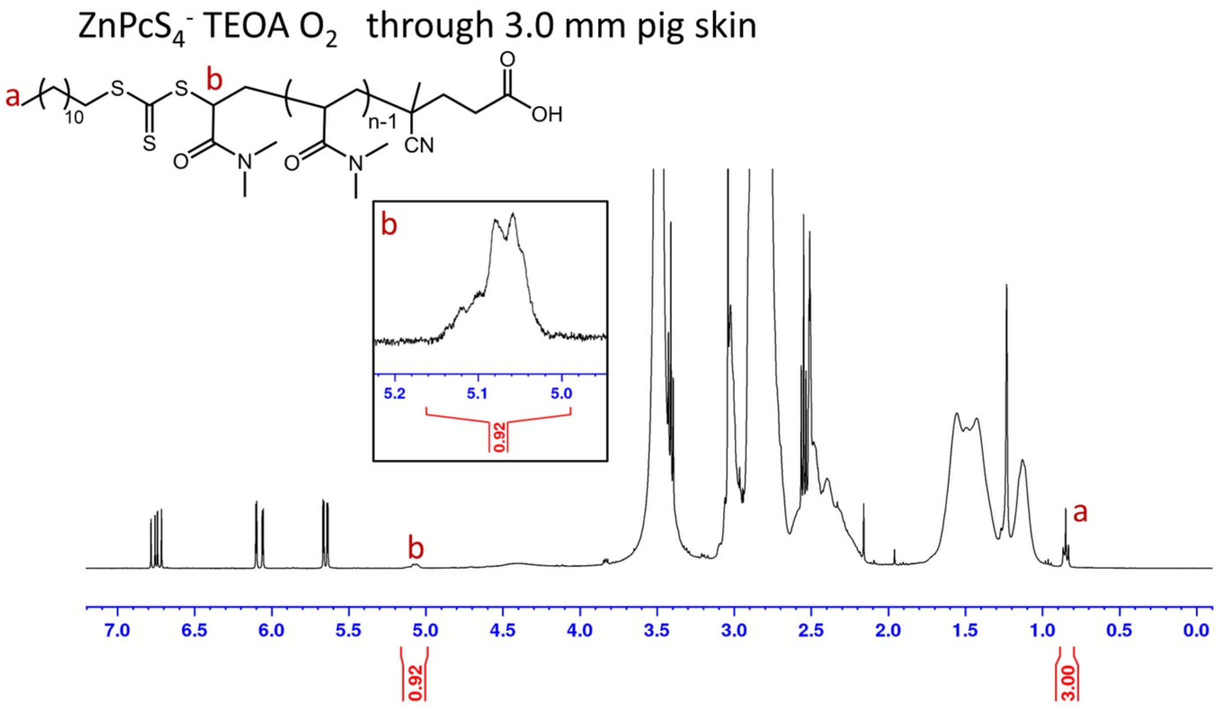

Figure S19. ^1H NMR spectroscopy of PDMA synthesized using CDTPA as the RAFT agent. Reactions were performed at room temperature under the irradiation of NIR light (λ_{max} = 730 nm; I = 60 mW cm^{-2}) passing through 3.0 mm pig skin (A) without prior deoxygenation (monomer conversion = 96%) in water at [DMA]/[water] = 50/50 (v/v) using a fixed reaction stoichiometry of [DMA]:[CDTPA]:[TEOA]:[$ZnPcS_4^-$] = 100:1:4:0.005 (50 ppm relative to monomer).

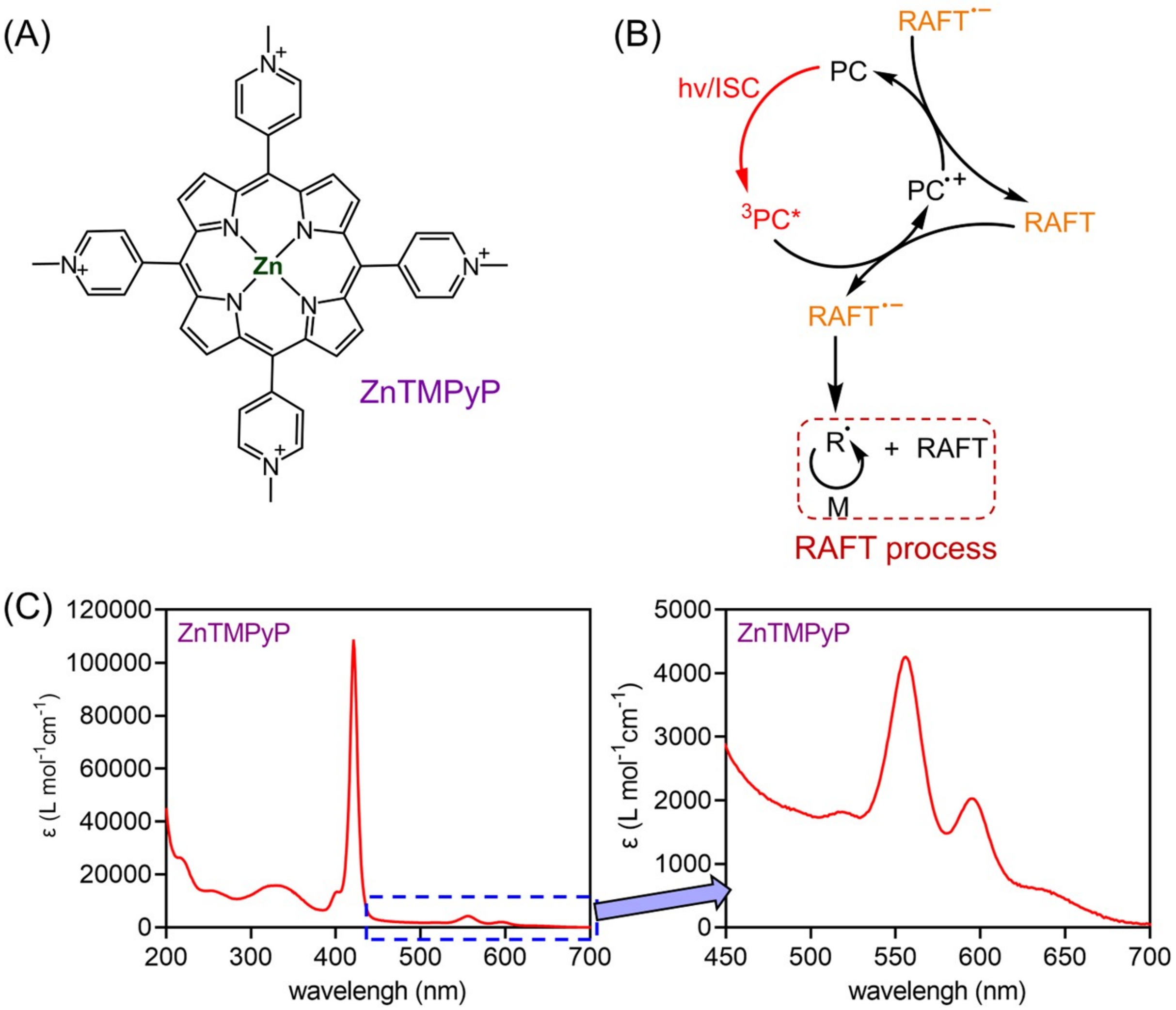

Figure S20. (A) Chemical structure of zinc meso-tetra(*N*-methyl-4-pyridyl) porphine tetrachloride (ZnTMPyP) (B) Mechanism of PET-RAFT polymerization via an oxidative quenching pathway (OQP) using ZnTMPyP as the PC. (C) UV-vis spectrum of ZnTMPyP in water.

Table S8. ZnTMPyP mediated photo-RAFT polymerization under the violet and green light irradiation passing through 3.0 mm pig skin.

#	Light irradiation wavelength (nm)	3.0 mm pig skin barrier	k_p^{app} (hour^{-1})	$M_{n, theo}$ (g mol^{-1})	$M_{n, GPC}$ (g mol^{-1})	$Đ$
1	Violet (405 nm)	no	6.30	14200	16100	1.13
2	Violet (405 nm)	yes	0	-	-	-
3	Green (530 nm)	no	0.93	13400	13600	1.15
4	Green (530 nm)	yes	0.07	9000	9600	1.18

Note: Reactions were irradiated by violet (λ_{max} = 405 nm; I_0 = 45 mW cm^{-2}) and green light (λ_{max} = 530 nm; I_0 = 72 mW cm^{-2}) passing through 3.0 mm pig skin for at room temperature without prior deoxygenation in water at [DMA]/[water] = 50/50 (v/v). A fixed reaction stoichiometry of [DMA]:[CTCPA]:[ZnTMPyP] = 200:1:0.01 (50 ppm relative to monomer) was used.

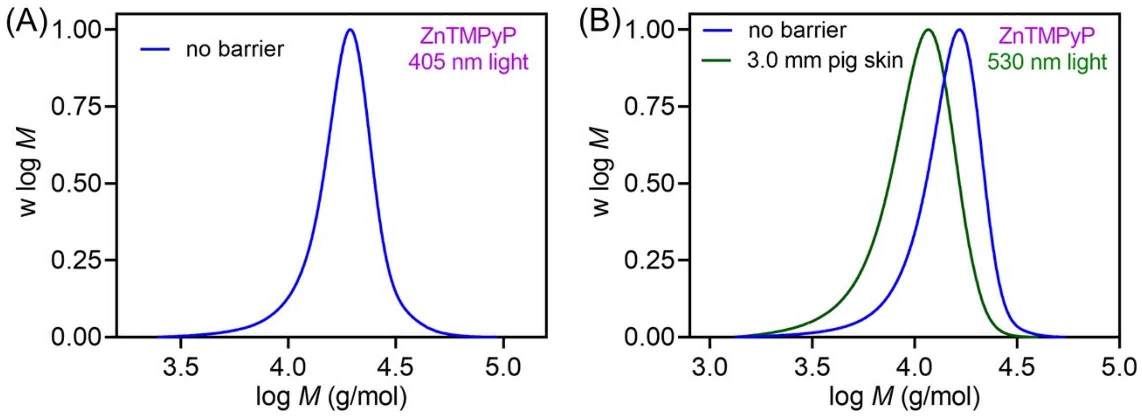

Figure S21. MWDs of synthesized PDMA by ZnTMPyP mediated photo-RAFT under violet (λ_{max} = 405 nm; I_0 = 45 mW cm^{-2}) and green (λ_{max} = 530 nm; I_0 = 72 mW cm^{-2}) light irradiation in the (A) absence and (B) presence of 3.0 mm pig skin barrier.

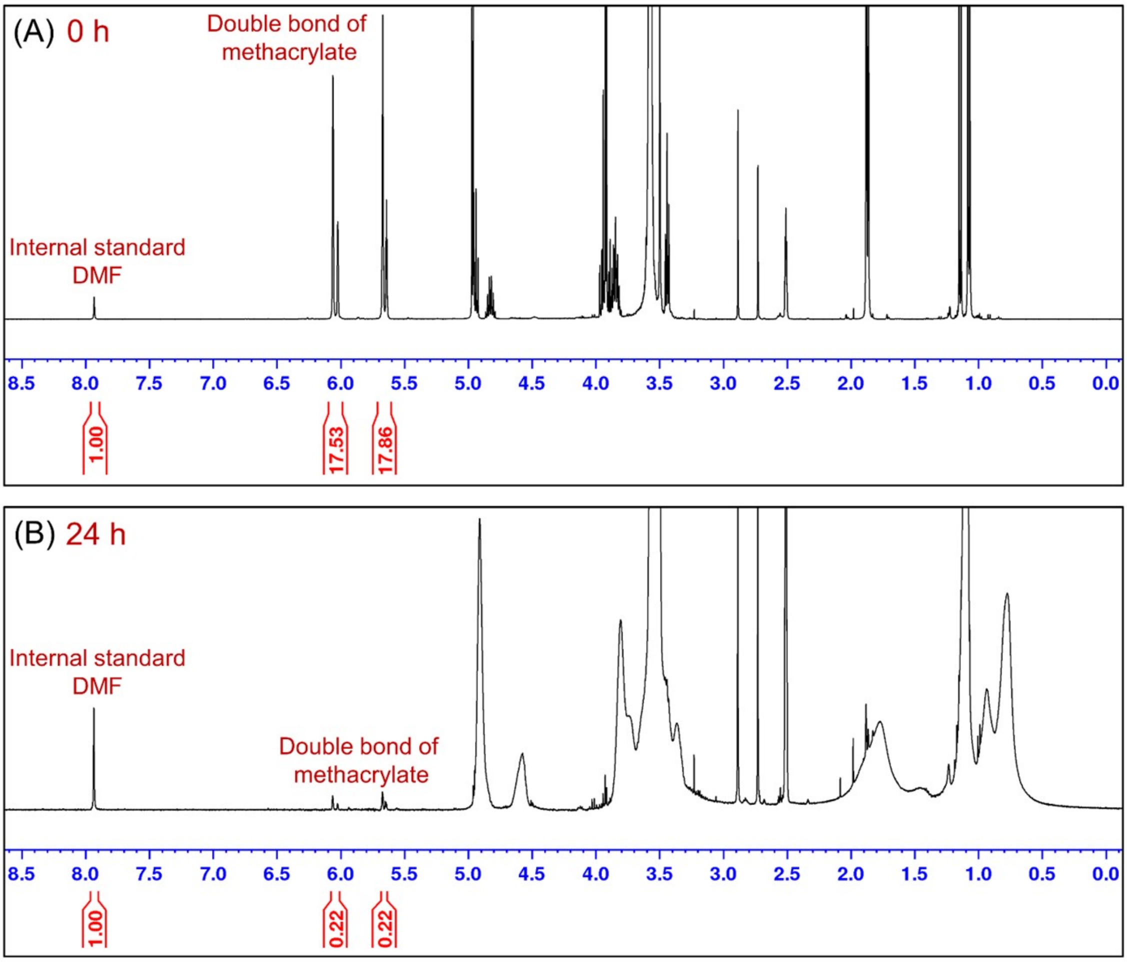

Figure S22. ^{1}H NMR spectra of crude photo-RAFT dispersion polymerization at different time points. Monomer conversions of dispersion photopolymerizations of HPMA were determined by ^{1}H NMR spectroscopy using DMF as the internal standard. Polymerizations were performed under the irradiation of NIR light for 24 hours without prior deoxygenation in water with a fixed reaction stoichiometry of [HPMA]:[PEG_{113}-CDTPA]:[TEOA]:[$ZnPcS_4^-$] = 350:1:4:0.02 and a total solids content of 20 wt%.

Table S9. Photo-RAFT dispersion polymerization catalyzed by $ZnPcS_4^-$ with various ratios of TEOA.

**#**	**[TEOA]:[RAFT]**	**α (%)**	$M_{n, theo}$ **(g mol⁻¹)**	$M_{n, GPC}$ **(g mol⁻¹)**	**Đ**
1	1	4	7400	10100	1.07
2	2	8	10800	11300	1.12
3	4	99	55300	73100	1.37
4	8	99	55300	74000	1.39

Note: Dispersion photopolymerizations were performed at room temperature under the irradiation of NIR light (λ_{max} = 730 nm; I = 60 mW cm^{-2}) for 24 hours without prior deoxygenation using HPMA as the monomer at a total solids content of 20 wt% in water. A fixed reaction stoichiometry of [HPMA]:[PEG_{113}-CDTPA]:[TEOA]:[$ZnPcS_4^-$] = 350:1:4:0.02 was used.

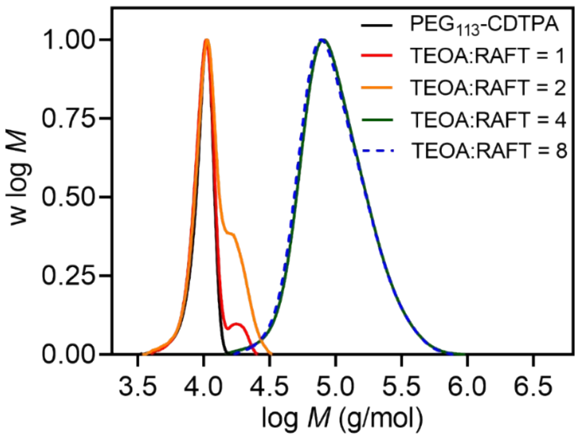

Figure S23. MWDs of synthesized PEG_{113}-*b*-PHPMA in the optimization experiments with various ratios (1, 2, 4, and 8) of TEOA related to RAFT agent using $ZnPcS_4^-$ as the photocatalyst. Polymerizations were performed under NIR light for 24 hours without prior deoxygenation in water with a fixed reaction stoichiometry of [HPMA]:[PEG_{113}-CDTPA]:[$ZnPcS_4^-$] = 350:1:0.02 and a total solids content of 20 wt%.

Table S10. Evolution of DLS data of self-assembled PEG113-*b*-PHPMA polymeric nanoparticles synthesized by $ZnPcS_4^-$ mediated photo-RAFT dispersion polymerization.

#	Irradiation time (hour)	Monomer conversion (%)	DLS D_h (nm)	DLS PDI
1	5	43	34	0.22
2	6	78	147	0.19
3	7	92	229	0.22

Note: Polymerizations were performed under NIR light (λ_{max} = 730 nm; 60 mW cm^{-2}) for 24 hours without prior deoxygenation using a fixed reaction stoichiometry of [HPMA]:[PEG_{113}-CDTPA]:[TEOA]:[$ZnPcS_4^-$] = 350:1:4:0.02 in water and a total solids content of 20 wt%.

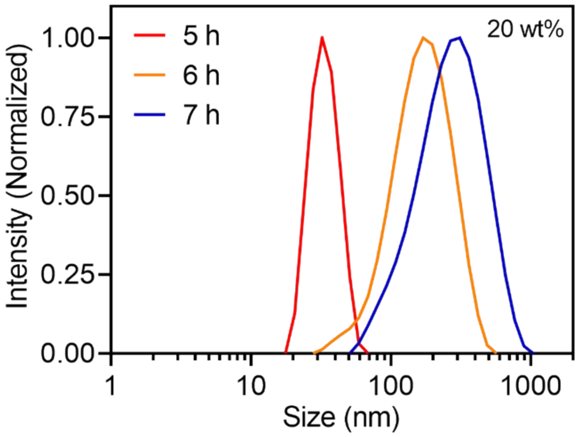

Figure S24. DLS derived intensity-based size distributions of self-assembled PEG113-*b*-PHPMA at different irradiation times (5, 6, and 7 hours) in **Table S8**.

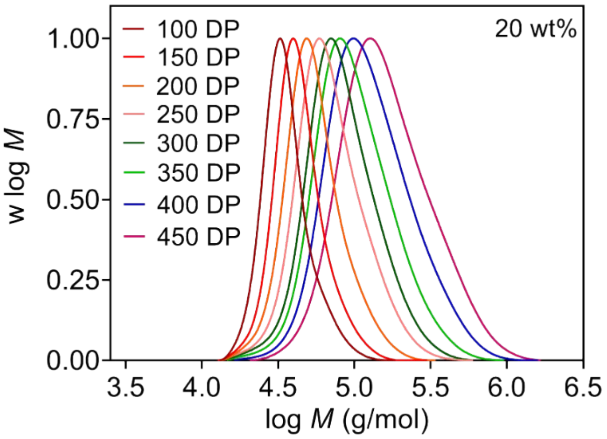

Figure S25. MWDs of PEG_{113}-*b*-PHPMA synthesized by $ZnPcS_4^-$ mediated photo-RAFT dispersion polymerization of HPMA. Polymerizations were conducted under NIR light irradiation (λ_{max} = 730 nm; 60 mW cm^{-2}) for 24 hours without prior deoxygenation in water with a fixed reaction stoichiometry of [PEG_{113}-CDTPA]:[TEOA]:[$ZnPcS_4^-$] = 1:4:0.02 and a total solids content of 20 wt%.

Table S11. $ZnPcS_4^-$ mediated photo-RAFT dispersion polymerization of HPMA with varying targeted DPs at a total solids content of 10 wt%.

#	DPs	α (%)	$M_{n, theo}$ (g mol^{-1})	$M_{n, GPC}$ (g mol^{-1})	$Đ$	Morphology (Major/Minor)
1	100	99	19700	26000	1.11	S
2	200	96	33100	42700	1.19	S
3	300	94	46000	55800	1.33	S
4	400	91	57900	69800	1.39	S

Note: Polymerizations were conducted under the irradiation of NIR light (λ_{max} = 730 nm; 60 mW cm^{-2}) for 24 hours without prior deoxygenation in water with a fixed reaction stoichiometry of [PEG_{113}-CDTPA]:[TEOA]:[$ZnPcS_4^-$] = 1:4:0.02 and a total solids content of 10 wt%.

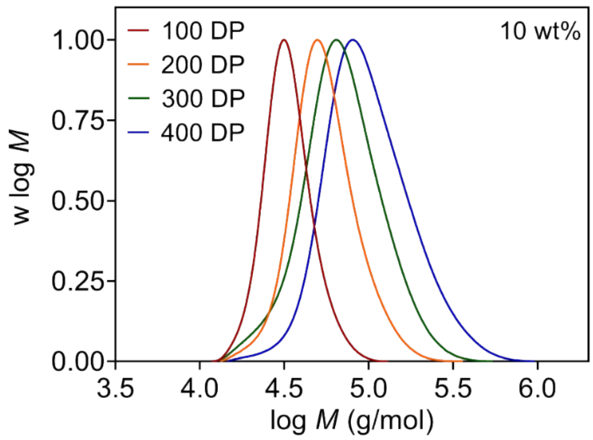

Figure S26. MWDs of PEG_{113}-*b*-PHPMA with various DPs synthesized by $ZnPcS_4^-$ mediated photo-RAFT dispersion polymerization of HPMA at a total solids content of 10 wt%.

Table S12. $ZnPcS_4^-$ mediated photo-RAFT dispersion polymerization of HPMA with varying target DPs at a total solids content of 15 wt%.

#	DPs	α (%)	$M_{n,\,theo}$ (g mol^{-1})	$M_{n,\,GPC}$ (g mol^{-1})	$Đ$	Morphology (Major/Minor)
1	100	100	19800	26200	1.11	S/W
2	200	98	33600	41700	1.19	W/S
3	300	96	46900	59800	1.32	W/S
4	400	92	58400	70500	1.41	V

Note: Polymerizations were conducted under the irradiation of NIR light (λ_{max} = 730 nm; 60 mW cm^{-2}) for 24 hours without prior deoxygenation in water with a fixed reaction stoichiometry of [PEG_{113}-CDTPA]:[TEOA]:[$ZnPcS_4^-$] = 1:4:0.02 and a total solids content of 15 wt%.

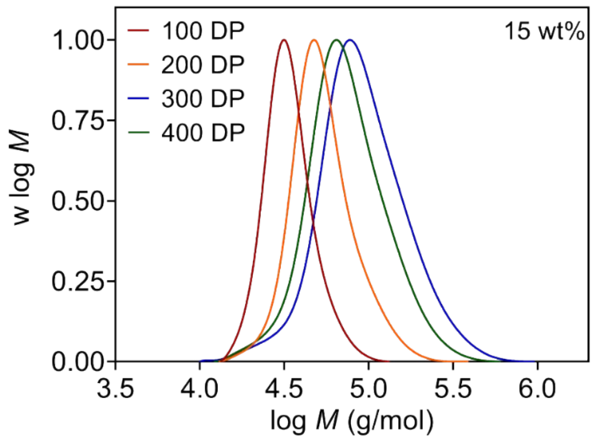

Figure S27. MWDs of PEG_{113}-*b*-PHPMA with various DPs synthesized by $ZnPcS_4^-$ mediated photo-RAFT dispersion polymerization of HPMA at a total solids content of 15 wt%.

Table S13. DLS analyses of self-assembled PEG_{113}-*b*-PHPMA polymeric nanoparticles synthesized by $ZnPcS_4^-$ mediated photo-RAFT dispersion polymerization.

#	Solid contents (wt%)	DPs	DLS D_h (nm)	DLS PDI	Morphology (Major/Minor)
1	10	100	36	0.12	S
2	10	200	52	0.08	S
3	10	300	59	0.05	S
4	10	400	69	0.01	S
5	15	100	35	0.14	S/W
6	15	200	74	0.04	W/S
7	15	300	95	0.13	W/S
8	15	400	158	0.06	V
9	20	100	38	0.21	S/W
10	20	200	156	0.52	W/S
11	20	300	252	0.23	W/V
12	20	400	408	0.23	V

Note: Polymerizations were performed under NIR light (λ_{max} = 730 nm; 60 mW cm^{-2}) for 24 hours without prior deoxygenation using a fixed reaction stoichiometry of [PEG_{113}-CDTPA]:[TEOA]:[$ZnPcS_4^-$] = 1:4:0.02 in water. 10, 15, and 20 wt% solid contents were used in the reaction with various targeted DPs including 100, 200, 300, and 400.

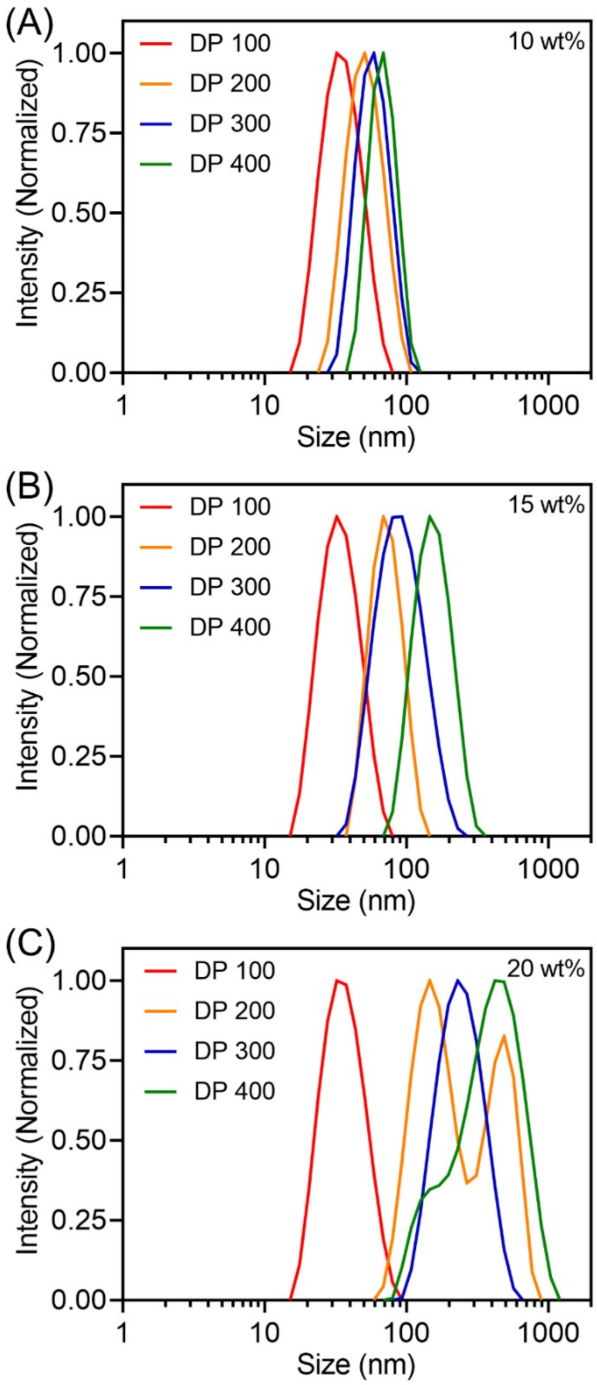

Figure S28. DLS derived intensity-based size distributions of self-assembled PEG_{113}-*b*-PHPMA in **Table S11** with different solid contents including (A) 10, (B) 15, and (C) 20 wt%.

Note: DLS was only used as an indicative measurement for non-spherical particles as DLS is only appropriate to be employed for spherical nanoparticles.

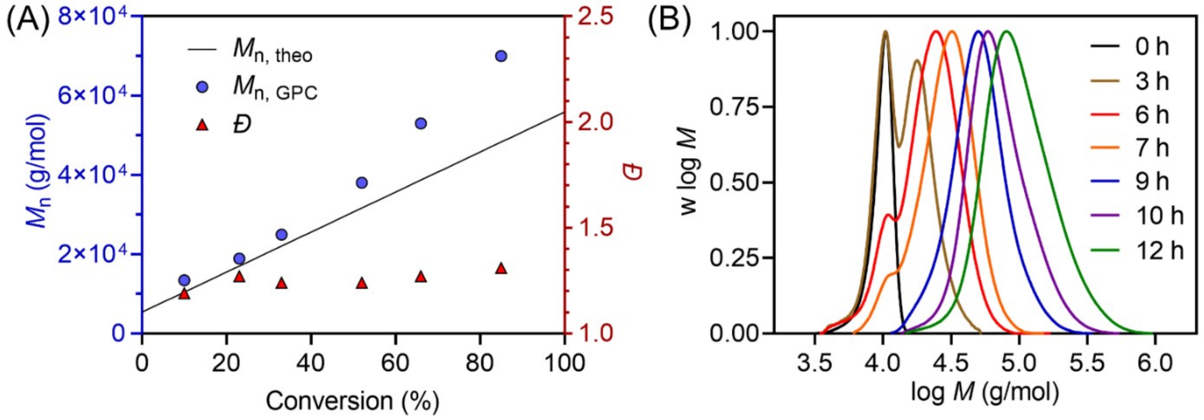

Figure S29. (A) Evolution of M_n and *Đ* *versus* monomer conversion obtained from $ZnPcS_4^-$ mediated photo-RAFT dispersion polymerization of HPMA under NIR light (λ_{max} = 730 nm; 60 mW cm^{-2}) irradiation passing through 6.0 mm pig skin using [HPMA]:[PEG_{113}-CDTPA]:[TEOA]:[$ZnPcS_{4-}$] = 350:1:4:0.02 at a total solids content of 20 wt%. (B) Evolution of normalized MWDs *versus* irradiation time from the reaction in (A).

DFT CALCULATIONS

Coordinates and energies of photocatalysts

The coordinates of photocatalysts are optimized at B3LYP-GD3BJ/6-311G* with the SMD-water solvation model (for C H N elements) and B3LYP-GD3BJ/LANL2TZ with the SMD-water solvation model (for Zn). All coordinates are exhibited as XYZ Cartesian coordinates. Single-point energy calculations were performed at B3LYP-GD3BJ/6-311+G** with the SMD-DMSO solvation model using the optimized coordinates. Zinc tetrasulfonated phthalocyanine ($ZnPcS_4^-$) was simplified to zinc phthalocyanine. All geometry optimizations and energies were calculated using the GAUSSIAN 09 D01 software package installed in the Katana high-performance computing clusters at the University of New South Wales.

PC

Ground state (PC) (-3447.155599)

C	1.11848300	2.78888400	0.08358300
N	0.00000000	2.01280400	0.18278000
C	-1.11848300	2.78888400	0.08358300
C	-0.70615900	4.18549900	-0.05540100
C	0.70615900	4.18549900	-0.05540100
N	-2.38834600	2.38834600	0.06253600
C	-2.78888400	1.11848300	0.08358300
N	-2.01280400	0.00000000	0.18278000
C	-2.78888400	-1.11848300	0.08358300
C	-4.18549900	-0.70615900	-0.05540100
C	-4.18549900	0.70615900	-0.05540100
N	2.38834600	2.38834600	0.06253600
C	4.18549900	0.70615900	-0.05540100
C	4.18549900	-0.70615900	-0.05540100
C	2.78888400	-1.11848300	0.08358300
N	2.01280400	0.00000000	0.18278000
C	2.78888400	1.11848300	0.08358300
N	2.38834600	-2.38834600	0.06253600
N	0.00000000	-2.01280400	0.18278000
C	1.11848300	-2.78888400	0.08358300
C	0.70615900	-4.18549900	-0.05540100
C	-0.70615900	-4.18549900	-0.05540100
C	-1.11848300	-2.78888400	0.08358300
N	-2.38834600	-2.38834600	0.06253600
Zn	0.00000000	0.00000000	0.70585600
C	5.37222400	1.42203600	-0.19095400
C	6.55846600	0.70203900	-0.31863500
C	6.55846600	-0.70203900	-0.31863500
C	5.37222400	-1.42203600	-0.19095400
C	-1.42203600	-5.37222400	-0.19095400
C	-0.70203900	-6.55846600	-0.31863500
C	0.70203900	-6.55846600	-0.31863500
C	1.42203600	-5.37222400	-0.19095400
C	-5.37222400	1.42203600	-0.19095400
C	-6.55846600	0.70203900	-0.31863500

C	-6.55846600	-0.70203900	-0.31863500
C	-5.37222400	-1.42203600	-0.19095400
C	1.42203600	5.37222400	-0.19095400
C	0.70203900	6.55846600	-0.31863500
C	-0.70203900	6.55846600	-0.31863500
C	-1.42203600	5.37222400	-0.19095400
H	5.37448300	2.50597800	-0.19589600
H	5.37448300	-2.50597800	-0.19589600
H	-2.50597800	-5.37448300	-0.19589600
H	2.50597800	-5.37448300	-0.19589600
H	-5.37448300	2.50597800	-0.19589600
H	-5.37448300	-2.50597800	-0.19589600
H	2.50597800	5.37448300	-0.19589600
H	-2.50597800	5.37448300	-0.19589600
H	-7.49850300	1.23263300	-0.42123100
H	-7.49850300	-1.23263300	-0.42123100
H	-1.23263300	7.49850300	-0.42123100
H	1.23263300	7.49850300	-0.42123100
H	7.49850300	1.23263300	-0.42123100
H	7.49850300	-1.23263300	-0.42123100
H	1.23263300	-7.49850300	-0.42123100
H	-1.23263300	-7.49850300	-0.42123100

Triplet state (^{3}PC) (-3447.116532)*

C	2.77802300	1.18208300	0.08434100
N	1.41519100	1.43113200	0.18921100
C	1.17911900	2.75742500	0.09076300
C	2.46521500	3.45660600	-0.04991000
C	3.46672900	2.46072200	-0.05055800
N	0.00000000	3.39889600	0.08192700
C	-1.17911900	2.75742500	0.09076300
N	-1.41519100	1.43113200	0.18921100
C	-2.77802300	1.18208300	0.08434100
C	-3.46672900	2.46072200	-0.05055800
C	-2.46521500	3.45660600	-0.04991000
N	3.38431300	0.00000000	0.05644000
C	3.46672900	-2.46072200	-0.05055800
C	2.46521500	-3.45660600	-0.04991000
C	1.17911900	-2.75742500	0.09076300
N	1.41519100	-1.43113200	0.18921100
C	2.77802300	-1.18208300	0.08434100
N	0.00000000	-3.39889600	0.08192700
N	-1.41519100	-1.43113200	0.18921100
C	-1.17911900	-2.75742500	0.09076300
C	-2.46521500	-3.45660600	-0.04991000
C	-3.46672900	-2.46072200	-0.05055800
C	-2.77802300	-1.18208300	0.08434100
N	-3.38431300	0.00000000	0.05644000
Zn	0.00000000	0.00000000	0.64379300
C	4.80891000	-2.80302300	-0.18410500
C	5.13155300	-4.15608200	-0.30833800
C	4.13694200	-5.14244500	-0.30761800
C	2.78741700	-4.80037100	-0.18196900

C	-4.80891000	-2.80302300	-0.18410500
C	-5.13155300	-4.15608200	-0.30833800
C	-4.13694200	-5.14244500	-0.30761800
C	-2.78741700	-4.80037100	-0.18196900
C	-2.78741700	4.80037100	-0.18196900
C	-4.13694200	5.14244500	-0.30761800
C	-5.13155300	4.15608200	-0.30833800
C	-4.80891000	2.80302300	-0.18410500
C	4.80891000	2.80302300	-0.18410500
C	5.13155300	4.15608200	-0.30833800
C	4.13694200	5.14244500	-0.30761800
C	2.78741700	4.80037100	-0.18196900
H	5.58346800	-2.04493200	-0.18942800
H	2.01761200	-5.56326200	-0.18667100
H	-5.58346800	-2.04493200	-0.18942800
H	-2.01761200	-5.56326200	-0.18667100
H	-2.01761200	5.56326200	-0.18667100
H	-5.58346800	2.04493200	-0.18942800
H	5.58346800	2.04493200	-0.18942800
H	2.01761200	5.56326200	-0.18667100
H	-4.41890200	6.18438500	-0.40800800
H	-6.17056700	4.44950400	-0.40873900
H	4.41890200	6.18438500	-0.40800800
H	6.17056700	4.44950400	-0.40873900
H	6.17056700	-4.44950400	-0.40873900
H	4.41890200	-6.18438500	-0.40800800
H	-4.41890200	-6.18438500	-0.40800800
H	-6.17056700	-4.44950400	-0.40873900

Anionic radical (PC$^{\bullet-}$) (-3447.280044)

C	2.76906200	1.18352000	0.07339200
N	1.43616600	1.42122500	0.15458000
C	1.18558700	2.78269500	0.07037700
C	2.45948100	3.46922000	-0.04445500
C	3.46145300	2.46536700	-0.04446000
N	0.00000000	3.38548400	0.04739100
C	-1.18558700	2.78269500	0.07037700
N	-1.43616600	1.42122500	0.15458000
C	-2.76906200	1.18352000	0.07339200
C	-3.46145300	2.46536700	-0.04446000
C	-2.45948100	3.46922000	-0.04445500
N	3.40429300	0.00000000	0.06363300
C	3.46145300	-2.46536700	-0.04446000
C	2.45948100	-3.46922000	-0.04445500
C	1.18558700	-2.78269500	0.07037700
N	1.43616600	-1.42122500	0.15458000
C	2.76906200	-1.18352000	0.07339200
N	0.00000000	-3.38548400	0.04739100
N	-1.43616600	-1.42122500	0.15458000
C	-1.18558700	-2.78269500	0.07037700
C	-2.45948100	-3.46922000	-0.04445500
C	-3.46145300	-2.46536700	-0.04446000
C	-2.76906200	-1.18352000	0.07339200

N	-3.40429300	0.00000000	0.06363300
Zn	0.00000000	0.00000000	0.57038200
C	4.81087700	-2.80050500	-0.15751300
C	5.15198700	-4.14574800	-0.26362600
C	4.15882300	-5.14319200	-0.26360300
C	2.81075800	-4.81773500	-0.15815000
C	-4.81087700	-2.80050500	-0.15751300
C	-5.15198700	-4.14574800	-0.26362600
C	-4.15882300	-5.14319200	-0.26360300
C	-2.81075800	-4.81773500	-0.15815000
C	-2.81075800	4.81773500	-0.15815000
C	-4.15882300	5.14319200	-0.26360300
C	-5.15198700	4.14574800	-0.26362600
C	-4.81087700	2.80050500	-0.15751300
C	4.81087700	2.80050500	-0.15751300
C	5.15198700	4.14574800	-0.26362600
C	4.15882300	5.14319200	-0.26360300
C	2.81075800	4.81773500	-0.15815000
H	5.57624400	-2.03220000	-0.16114700
H	2.05227800	-5.59285700	-0.16177100
H	-5.57624400	-2.03220000	-0.16114700
H	-2.05227800	-5.59285700	-0.16177100
H	-2.05227800	5.59285700	-0.16177100
H	-5.57624400	2.03220000	-0.16114700
H	5.57624400	2.03220000	-0.16114700
H	2.05227800	5.59285700	-0.16177100
H	-4.45316300	6.18392300	-0.34851100
H	-6.19450800	4.43246000	-0.34885400
H	4.45316300	6.18392300	-0.34851100
H	6.19450800	4.43246000	-0.34885400
H	6.19450800	-4.43246000	-0.34885400
H	4.45316300	-6.18392300	-0.34851100
H	-4.45316300	-6.18392300	-0.34851100
H	-6.19450800	-4.43246000	-0.34885400

Cationic radical ($PC^{\bullet+}$) (-3446.975349)

C	1.11544400	2.78176600	0.09685700
N	0.00000000	2.00628500	0.21504400
C	-1.11544400	2.78176600	0.09685700
C	-0.70424200	4.18211900	-0.06245200
C	0.70424200	4.18211900	-0.06245200
N	-2.38612500	2.38612500	0.07370400
C	-2.78176600	1.11544400	0.09685700
N	-2.00628500	0.00000000	0.21504400
C	-2.78176600	-1.11544400	0.09685700
C	-4.18211900	-0.70424200	-0.06245200
C	-4.18211900	0.70424200	-0.06245200
N	2.38612500	2.38612500	0.07370400
C	4.18211900	0.70424200	-0.06245200
C	4.18211900	-0.70424200	-0.06245200
C	2.78176600	-1.11544400	0.09685700
N	2.00628500	0.00000000	0.21504400
C	2.78176600	1.11544400	0.09685700

N	2.38612500	-2.38612500	0.07370400
N	0.00000000	-2.00628500	0.21504400
C	1.11544400	-2.78176600	0.09685700
C	0.70424200	-4.18211900	-0.06245200
C	-0.70424200	-4.18211900	-0.06245200
C	-1.11544400	-2.78176600	0.09685700
N	-2.38612500	-2.38612500	0.07370400
Zn	0.00000000	0.00000000	0.76920800
C	5.35815700	1.42282800	-0.21325600
C	6.54763400	0.69882100	-0.35562900
C	6.54763400	-0.69882100	-0.35562900
C	5.35815700	-1.42282800	-0.21325600
C	-1.42282800	-5.35815700	-0.21325600
C	-0.69882100	-6.54763400	-0.35562900
C	0.69882100	-6.54763400	-0.35562900
C	1.42282800	-5.35815700	-0.21325600
C	-5.35815700	1.42282800	-0.21325600
C	-6.54763400	0.69882100	-0.35562900
C	-6.54763400	-0.69882100	-0.35562900
C	-5.35815700	-1.42282800	-0.21325600
C	1.42282800	5.35815700	-0.21325600
C	0.69882100	6.54763400	-0.35562900
C	-0.69882100	6.54763400	-0.35562900
C	-1.42282800	5.35815700	-0.21325600
H	5.36009200	2.50616700	-0.21874200
H	5.36009200	-2.50616700	-0.21874200
H	-2.50616700	-5.36009200	-0.21874200
H	2.50616700	-5.36009200	-0.21874200
H	-5.36009200	2.50616700	-0.21874200
H	-5.36009200	-2.50616700	-0.21874200
H	2.50616700	5.36009200	-0.21874200
H	-2.50616700	5.36009200	-0.21874200
H	-7.48498600	1.23079200	-0.46953300
H	-7.48498600	-1.23079200	-0.46953300
H	-1.23079200	7.48498600	-0.46953300
H	1.23079200	7.48498600	-0.46953300
H	7.48498600	1.23079200	-0.46953300
H	7.48498600	-1.23079200	-0.46953300
H	1.23079200	-7.48498600	-0.46953300
H	-1.23079200	-7.48498600	-0.46953300

Coordinates and energies of RAFT agent, TEOA, and O_2

The coordinates of RAFT agent, TEOA, and O_2 are optimized at B3LYP-GD3BJ/6-311G* with the SMD-water solvation model exhibited as XYZ Cartesian coordinates. Single-point energy calculations were performed at B3LYP-GD3BJ/6-311+G** with the SMD-water solvation model using the optimized coordinates. All geometry optimizations and energies were calculated using the GAUSSIAN 09 D01 software package installed in the Katana high-performance computing clusters at the University of New South Wales.

CTCPA

Ground state (CTCPA) (-1939.523066)

S	-0.33247500	0.31936600	1.30579300
S	0.61714400	-1.77126900	-0.71970000
C	2.31159700	-1.28619900	-0.10000100
C	2.37177600	-1.47294200	1.34852000
N	2.50562700	-1.67637400	2.47724700
C	3.23755300	-2.32814700	-0.75902400
H	3.21625200	-2.20359600	-1.84221100
H	4.25957200	-2.17926600	-0.41147000
H	2.93955300	-3.34595700	-0.50559900
C	2.65799900	0.15068000	-0.53271100
H	2.64972300	0.16509600	-1.62359100
H	1.88431500	0.83379400	-0.18984100
C	-0.55338900	-0.64585700	-0.00638500
C	4.01165700	0.62192800	-0.00979500
H	4.07244700	0.53833500	1.07973800
H	4.83695000	0.02581000	-0.40572400
C	4.27971800	2.05916100	-0.36144100
O	3.52704700	2.79797200	-0.96117600
O	5.48946800	2.45774300	0.08020200
H	5.61946200	3.39021300	-0.15781100
S	-2.03020700	-0.78751900	-0.95164700
C	-3.25354800	0.15070300	0.03733700
H	-3.28926600	-0.28289700	1.03430400
H	-2.91294200	1.18161800	0.10812600
C	-4.59707400	0.05130400	-0.67628100
H	-4.92877700	-0.99005600	-0.74255500
H	-4.53882400	0.42526000	-1.70043000
C	-5.71887900	0.80676700	-0.00753800
O	-6.77109600	1.06291200	-0.55345700
O	-5.45503000	1.15017700	1.26409700
H	-6.22681400	1.61221200	1.63179200

Anionic radical (CTCPA•−) (-1939.636429)

S	1.39017800	-2.39571200	1.62770700
S	2.57029500	-1.04783400	-0.84787100
C	2.30080900	0.82629100	-0.43368800
C	2.95663300	1.07431800	0.83898100
N	3.48539800	1.28319300	1.84772600

C	3.00669500	1.59767900	-1.55191300
H	2.46962200	1.44619400	-2.48932100
H	3.03268100	2.66729100	-1.33249700
H	4.03638700	1.25729100	-1.67863000
C	0.79878400	1.11325900	-0.33308500
H	0.33923800	0.81337000	-1.27579700
H	0.37768700	0.47843700	0.44485900
C	1.29773500	-1.92500300	-0.02028700
C	0.46955700	2.57128400	-0.03044700
H	0.98964200	2.92130200	0.86851100
H	0.77972300	3.23936000	-0.83667400
C	-1.00009000	2.78331800	0.20891000
O	-1.83675200	1.91311800	0.33797600
O	-1.30910000	4.09419300	0.28474700
H	-2.26036400	4.18139700	0.46024600
S	-0.14637900	-2.25921900	-1.02649300
C	-1.55931700	-1.73607000	0.04307900
H	-1.94714500	-2.59794800	0.58358500
H	-1.16308800	-1.03017700	0.76844200
C	-2.63410400	-1.09229700	-0.82192900
H	-3.03979900	-1.80198200	-1.54944400
H	-2.21535000	-0.26877900	-1.40545100
C	-3.81673000	-0.51745300	-0.08453900
O	-4.75140300	0.03031100	-0.63432700
O	-3.75261200	-0.65798600	1.25156700
H	-4.54342900	-0.25290600	1.64441000

TEOA

Ground state (TEOA)(-518.276192)

N	-0.00575100	-0.00285000	0.30768300
C	0.52997100	-1.31047300	0.70983700
C	0.85766100	1.10777700	0.73298900
C	-1.39819600	0.18075000	0.73804800
C	1.47383600	-1.93385200	-0.29828600
C	0.95775100	2.23027800	-0.27869800
C	-2.42251400	-0.28292500	-0.27636600
H	-0.30172600	-2.00465000	0.82127200
H	1.02568200	-1.25208100	1.69216100
H	1.86905000	0.72932500	0.87454100
H	0.53432700	1.51252100	1.70493500
H	-1.57707800	1.24372900	0.89417300
H	-1.58526900	-0.31392900	1.70449000
H	0.96712000	-2.01594500	-1.26643300
H	1.31603100	1.82425000	-1.23153300
H	-2.24400900	0.23166300	-1.22732900
O	-2.33878400	-1.70381200	-0.46027900
H	-2.96634100	-1.94758900	-1.15049000
H	-3.42346000	-0.00944200	0.08165800
H	1.73179500	-2.94607000	0.03906400
O	2.66531300	-1.14444500	-0.43076500
H	3.19236200	-1.52640800	-1.14213700
O	-0.31992200	2.85804500	-0.46116600

H	-0.23223700	3.50107700	-1.17418400
H	1.69010000	2.96585700	0.07857200

Cationic radical (TEOA$^{\bullet+}$)(-518.094921)

N	-0.00111800	0.00123800	0.89780300
C	-1.34468400	0.53601600	0.87610900
H	-1.90553800	0.06579400	1.68906200
H	-1.28590700	1.60609900	1.05397000
C	0.20958600	-1.42855700	0.86927000
H	0.89636000	-1.68397200	1.68099500
H	-0.74593500	-1.91689000	1.04022600
C	1.12984400	0.90000900	0.89521800
H	0.98349800	1.62670500	1.69920800
H	2.02854900	0.32200300	1.09267300
C	0.81738000	-1.96504800	-0.45082800
H	0.27034100	-1.57436300	-1.31052900
C	1.32319600	1.68638800	-0.42609800
H	1.31371800	1.00625900	-1.27886600
C	-2.12229000	0.28865400	-0.43988900
H	-1.51153000	0.56861500	-1.29943100
H	-2.98148300	0.96032300	-0.38935200
H	2.31752600	2.13015200	-0.34433400
H	0.67202600	-3.04635600	-0.40751800
O	-2.61706500	-1.03279100	-0.56775200
H	-2.03271700	-1.54109900	-1.14034600
O	0.38983400	2.73786300	-0.60013400
H	-0.28660600	2.46531300	-1.22972500
O	2.20766600	-1.72083600	-0.57193000
H	2.35385400	-0.97591500	-1.16482500

Oxygen

Triplet state (O_2) (-150.371874)

O	0.00000000	0.00000000	0.60227800
O	0.00000000	0.00000000	-0.60227800

Singlet state ($^1O_2$) (-150.312597)

O	0.00000000	0.00000000	0.60251800
O	0.00000000	0.00000000	-0.60251800

Anionic radical ($O_2^{\bullet-}$) (-150.519608)

O	0.00000000	0.00000000	0.67344900
O	0.00000000	0.00000000	-0.67344900

REFERENCES

1. E. Corey and W. C. Taylor, *J. Am. Chem. Soc.*, 1964, **86**, 3881-3882.
2. R.-h. Liu, S.-y. Fu, H.-y. Zhan and L. A. Lucia, *Ind. Eng. Chem. Res.*, 2009, **48**, 9331-9334.
